# Modelling the potential effectiveness of hepatitis C screening and treatment strategies during pregnancy in Egypt and Ukraine

**DOI:** 10.1016/j.jhep.2022.12.032

**Published:** 2023-01-18

**Authors:** Nadia Hachicha-Maalej, Intira Jeannie Collins, Anthony E. Ades, Karen Scott, Ali Judd, Aya Mostafa, Elizabeth Chappell, Manal Hamdy-El-Sayed, Diana Gibb, Sarah Pett, Eugènia Mariné-Barjoan, Alla Volokha, Yazdan Yazdanpanah, Sylvie Deuffic-Burban

**Affiliations:** 1 Université Paris Cité and Université Sorbonne Paris Nord, Inserm, IAME, F-75018 Paris, France; 2 MRC Clinical Trials Unit at UCL, Institute of Clinical Trials & Methodology, London, UK; 3 Population Health Sciences, University of Bristol Medical School, Bristol, UK; 4 Department of Community, Environmental, and Occupational Medicine, Faculty of Medicine, Ain Shams University, Cairo, Egypt; 5 Institute for Global Health, University College London, London, UK; 6 Département de Santé Publique/Pôle Référence Hépatite C, CHU Nice, Nice, France; 7 Department of Paediatric Infectious Diseases and Paediatric Immunology, Shupyk National Healthcare University of Ukraine, Kyiv, Ukraine; 8 Service de Maladies Infectieuses et Tropicales, Hôpital Bichat-Claude Bernard, F-75018 Paris, France

**Keywords:** HCV screening, DAA treatment, pregnancy, modelling, effectiveness, vertical transmission

## Abstract

**Background & Aims:**

HCV test and treat campaigns currently exclude pregnant women. Pregnancy offers a unique opportunity for HCV screening and to potentially initiate direct-acting antiviral treatment. We explored HCV screening and treatment strategies in two lower middle-income countries with high HCV prevalence, Egypt and Ukraine.

**Methods:**

Country-specific probabilistic decision models were developed to simulate a cohort of pregnant women. We compared five strategies: S0, targeted risk-based screening and deferred treatment (DT) to after pregnancy/breastfeeding; S1, World Health Organization (WHO) risk-based screening and DT; S2, WHO risk-based screening and targeted treatment (treat women with risk factors for HCV vertical transmission [VT]); S3, universal screening and targeted treatment during pregnancy; S4, universal screening and treatment. Maternal and infant HCV outcomes were projected.

**Results:**

S0 resulted in the highest proportion of women undiagnosed: 59% and 20% in Egypt and Ukraine, respectively, with 0% maternal cure by delivery and VT estimated at 6.5% and 7.9%, respectively. WHO risk-based screening and DT (S1) increased the proportion of women diagnosed with no change in maternal cure or VT. Universal screening and treatment during pregnancy (S4) resulted in the highest proportion of women diagnosed and cured by delivery (65% and 70%, respectively), and lower levels of VT (3.4% and 3.6%, respectively).

**Conclusions:**

This is one of the first models to explore HCV screening and treatment strategies in pregnancy, which will be critical in informing future care and policy as more safety/efficacy data emerge. Universal screening and treatment in pregnancy could potentially improve both maternal and infant outcomes.

## Introduction

In 2019, 56.8 million people were estimated to be living with chronic hepatitis C infection worldwide, with the majority of them in lower middle-income countries (LMICs).^1^ HCV treatment has evolved rapidly in the last 10 years, with the emergence of several well-tolerated and highly efficacious short-course direct-acting antivirals (DAAs) that can cure chronic hepatitis C, leading many countries to commit to the World Health Organization (WHO) goals for the elimination of hepatitis C by 2030. However, access to testing remains limited and WHO recommendations to screen populations with a high HCV prevalence or who have a history of HCV risk exposure/behaviour^2^ have not been well implemented; screening is often only targeted at key populations considered most at risk. It is estimated that only 23% of HCV RNA-positive individuals are diagnosed globally (or 15% in LMICs).^3^

Chronic hepatitis C is a major problem in women of child-bearing age, with increased risks of poor outcomes in pregnancy, including higher risk of intrahepatic cholestasis, preterm birth, and low birth weight.^4,5^ There is also the estimated 5% risk of vertical transmission (VT) of HCV, which increases further among women with unsuppressed HIV coinfection^6^ or high levels of HCV RNA.^7,8^

Pregnant women are currently excluded from HCV treatment programs.^9^ Global estimates suggest that in 2018-2019 there were an estimated 14.86 million women of childbearing age (age 15–49 years) and 3.26 million children aged ≤18 living with HCV.^10,11^ Pregnant women and paediatric populations are at risk of being left behind as DAAs are not approved for use during pregnancy or lactation or in early childhood. In LMIC settings, the duration of breastfeeding is often long and women may have pregnancies in quick succession, which can lead to long delays before women are eligible for treatment, increasing the risk of disease progression, VT and loss to follow-up.^12,13^

Egypt and Ukraine are two LMICs with high HCV prevalence, with a generalised epidemic in Egypt linked to treatment campaigns against schistosomiasis with unsafe intravenous injections during the 1970s–80s,^14^ and a concentrated epidemic in Ukraine associated with injection drug use and HIV.^15^ In both contexts, elimination of HCV may only be achieved if strategies include pregnant women.^9^

Whilst findings from the first DAA trials in non-pregnant adults were reported a decade ago, there is only one published pharmacokinetic study on DAAs in pregnant women: a study of the non-pangenotypic sofosbuvir/ledipasvir regimen in eight pregnant women (all treated in the second/third trimester) who all achieved HCV cure, with no VT or safety issues.^16^ An ongoing sofosbuvir/velpatasvir pharmacokinetic study in 10 women, with treatment initiated from 14 to 22 weeks’ gestation, is actively recruiting and will report in 02/2023,^17^ and a single-arm safety study (n = 100, USA) of third trimester sofosbuvir/velpatasvir, conducted at the same site, will report in 2025.^18^ If DAAs are found to be safe and efficacious in pregnancy, it provides a unique opportunity to screen, initiate treatment and cure women during pregnancy, which will potentially reduce the risk of VT and adverse maternal and infant outcomes associated with HCV.^16,19^ Recent surveys of pregnant women in Egypt and Ukraine have found high acceptability of routine HCV screening in antenatal care and likely high uptake of DAAs if approved for use in pregnancy.^20^ Assuming that HCV treatment during pregnancy is safe, our aim was to explore the potential impact of different HCV screening and treatment strategies on maternal and infant HCV outcomes in Egypt and Ukraine using a Markov model developed and applied to each setting.

## Materials and methods

### Study design

We designed a probabilistic decision model to evaluate the effectiveness of five different screening and treatment strategies during pregnancy on maternal HCV status (including diagnosis in pregnancy and cure by delivery) and VT based on infant HCV status at 6 months of age, in a hypothetical cohort of pregnant women and their infants in Egypt and Ukraine. The reference strategy is the standard of care (SOC) of targeted risk-based screening and deferred treatment to after delivery and cessation of breastfeeding (S0). This is compared to four alternative strategies outlined in Table 1, which include WHO recommended risk-based screening or universal screening combined with deferred or universal treatment.

Screening is defined as HCV antibody testing in serum, with all positive results then being tested for HCV RNA.

### Model structure

**Fig. 1:**
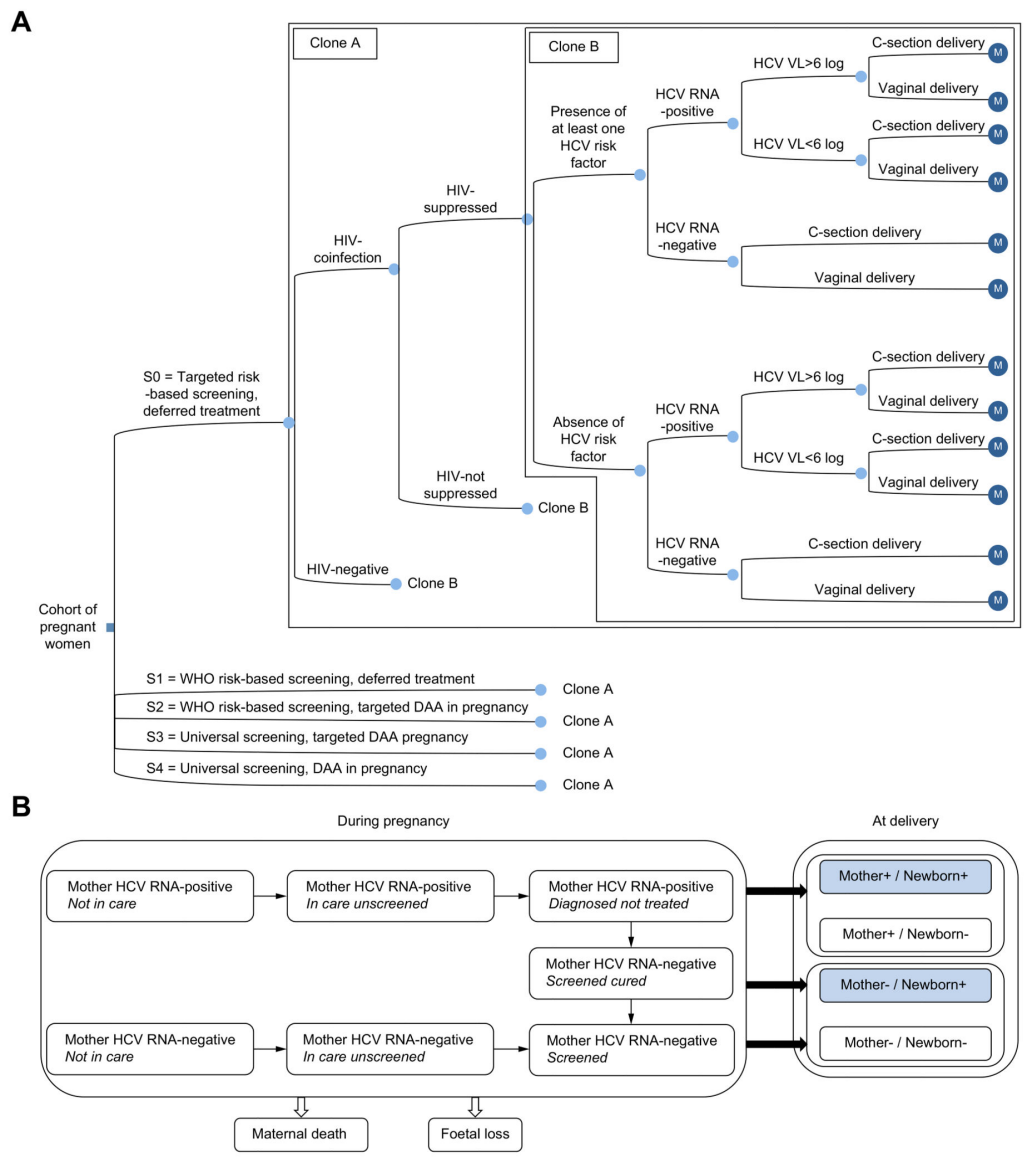
Model structure. (A) Decision tree stratified according to the different screening and treatment strategies and according to the characteristics of pregnant women; The five modelled strategies are shown at the decision node, indicated by a square. Chance nodes, at which events occur, are indicated by circles; Markov nodes are indicated by purple circle containing an ‘M’. (B) Markov-based model: trajectory of women in antenatal care and pregnancy outcomes for mother and child related to HCV infection. Maternal outcomes are calculated at delivery, infant’s outcome is calculated at 6 months of age. DAA, direct-acting antiviral; VL, viral load; WHO, World Health Organization.

A decision tree was combined with a Markov-based model to simulate the trajectory of pregnant women and their newborns, from entry into antenatal care until the end of the pregnancy for each strategy and setting. The decision tree was stratified on women’s characteristics impacting the probability of HCV screening and treatment (Fig. 1A). The main characteristics considered were: HIV status, women living with HIV were stratified by HIV RNA viral suppression status (defined as <1,000 copies/ml); the presence of ≥1 risk factor for HCV infection (Table S1); HCV RNA status, HCV RNA-positive individuals were stratified by HCV viral load (≥ or <6 log IU/ml); finally, the mode of delivery (planned c-section or not) (Fig. 1A).

The Markov-based model then simulated for each woman the different events that may occur during pregnancy (Fig. 1B). Briefly, at each monthly cycle there is the probability of: first presentation to antenatal care (ANC), uptake of HCV screening, uptake of DAA treatment among HCV RNA-positive women (offered from the start of the third trimester), HCV cure among women receiving DAAs, and HCV RNA-positive infants. The Markov model incorporates the probability of a spontaneous HCV clearance in infants, and the figures we provide relate to a risk of HCV RNA positivity at 6 months of age (*i.e*. VT rate net of clearance at 6 months).

In this model, we assume that women remain in care from first presentation to ANC through to delivery and that HCV screening is only offered at first presentation. Women who test PCR positive will have a probability of initiating 8-week treatment from the start of the third trimester. Late presentation to ANC, diagnosis of HCV and initiation of DAAs in the latter part of the third trimester assumes a reduced efficacy in preventing VT.

Among women who receive DAA treatments in pregnancy we assume rapid virologic response (within 4 weeks) as reported in the general adult population,^21,22^ which is assumed to help reduce the risk of VT.

At each cycle, women were at risk of death and foetal loss. We assumed that foetal loss led to entry into ANC for women who were not yet linked to care, and therefore eligibility for HCV screening and treatment. Delivery could take place from the sixth month of pregnancy onwards. This led us to calculate maternal outcomes among all HCV RNA-positive women who have started a pregnancy and paediatric outcomes among HCV RNA-positive women who have given birth.

### Input parameters

#### Study population

The study population consisted of the total number of pregnancies per year: 3 million in Egypt and 570,000 in Ukraine^23–26^ (supplementary information). Values for the key model parameters are given in Table 2; the complete list of parameters is given in Table S2.

Country-specific HIV prevalence and antiretroviral coverage rates were derived from 2020 UNAIDS data.^27,28^ Among women living with HIV, 24% (Egypt) and 86% (Ukraine) were assumed to be virally suppressed and to have the same risk of HCV VT as HCV-monoinfected women (supplementary information).^27,28^ Among HIV-negative women, 89% (Egypt) and 79% (Ukraine) have at least one risk factor for HCV infection. HCV prevalence was determined according to the presence/absence of at least one risk factor for HCV infection^11,20,29–31^ (supplementary information for detailed calculation).

High HCV viral load ≥6 log IU/ml is considered a risk factor of VT.^7^ HIV viral load is a risk factor for high HCV viral load.^7^ Based on data from the ALHICE study, the proportion of women with high HCV viral load was: 44% among unsuppressed HIV-positive *vs*. 28% in HIV-negative/HIV virally suppressed.^7^

We assumed that the proportion of women with suppressed HIV coinfection with high HCV viral load was the same as in the HIV-negative population. Women with uncontrolled HIV coinfection would have higher risk of high HCV viral load.

Data on access to ANC were derived from DHS surveys in Egypt and Ukraine.^32,33^ Data on maternal mortality, foetal mortality, mode of delivery and duration of pregnancies are detailed in the supplementary information.

#### Screening and treatment assumptions

The assumptions for uptake of HCV screening and DAA treatment are shown in Table 3 and are based on recent acceptability studies surveying pregnant and post-partum women in Egypt and Ukraine,^20^ except the uptake of HCV screening in Egypt for strategy S0 (current SOC) which was based on expert opinion. For strategy S0, uptake of screening ranged between 10% (no HCV infection risk factors and no planned c-section) and 60% (≥1 HCV risk factors and planned c-section) in Egypt, whereas it ranged between 75% (no HCV risk factors) and 87% (among HIV-positive women) in Ukraine.

In strategies S1 and S2, HCV screening increased to 88% in Egypt and 95% in Ukraine among women with HCV risk factors; in strategies S3 and S4, with universal screening, uptake was high across all groups irrespective of risk factors, based on the acceptability surveys. Regarding treatment uptake in pregnancy, this is assumed to be the same among women with risk factors for VT (strategies S2 and S3 with targeted treatment) as in all HCV RNA-positive women (S4, universal treatment) based on the acceptability survey.

Regarding efficacy of DAA treatment in pregnancy, we assumed 95% of pregnant women would achieve a rapid virologic response within 4 weeks.^21,22^

#### Vertical transmission

The probability of VT was introduced into the model as different functions of a common underlying set of parameters taking into account the characteristics of the population, *i.e*. the mother’s HIV status and HCV RNA-viral load group (transmission risk factors). This was obtained from the analysis of the data on infants born to HCV antibody-positive mothers using a Bayesian multi-parameter evidence synthesis.^8^ This analysis simultaneously estimated overall VT rates and rates net of clearance after applying different rates of HCV clearance at different ages of the child obtained from another study on the same data set.^34^ As clearance rate declines rapidly over the first 6 months and, as it appears that the meta-analysis VT rate of 5.8% represents a VT rate net of clearance at just under 6 months,^6^ we also applied a clearance rate at 6 months to overall VT risks. Specifically, in our model we used transmission equations consisting of transmission rates at different stages of pregnancy, combined with odds ratios for transmission risk factors, all multiplied by a clearance rate at 6 months (supplementary information).

Considering that VT mainly occurs late in utero or at delivery,^8^ we assumed that treatment initiation at least 4 weeks before delivery followed by a rapid virologic response would reduce VT by 80%; otherwise the reduction would only be 5%.

### Sensitivity analysis

Extensive sensitivity analyses were conducted to explore the impact of uncertainties around the input parameters and model assumptions. First, we varied values of each input parameter that may change our conclusions with the lower and upper bounds of its uncertainty interval based on the literature or set to +/-10% if not available (Table S3). To explore a lower impact of treatment initiation on VT reduction, we reduced the efficacy assumptions to 72% (*vs*. 80%) when treatment is initiated at least 4 weeks before delivery and 0% (*vs*. 5%) otherwise. Second, we simultaneously varied model parameter values from appropriate probability distributions across 10,000 simulations (supplementary information and Table S3).

## Results

### Baseline analysis

#### Egypt

**Fig. 2:**
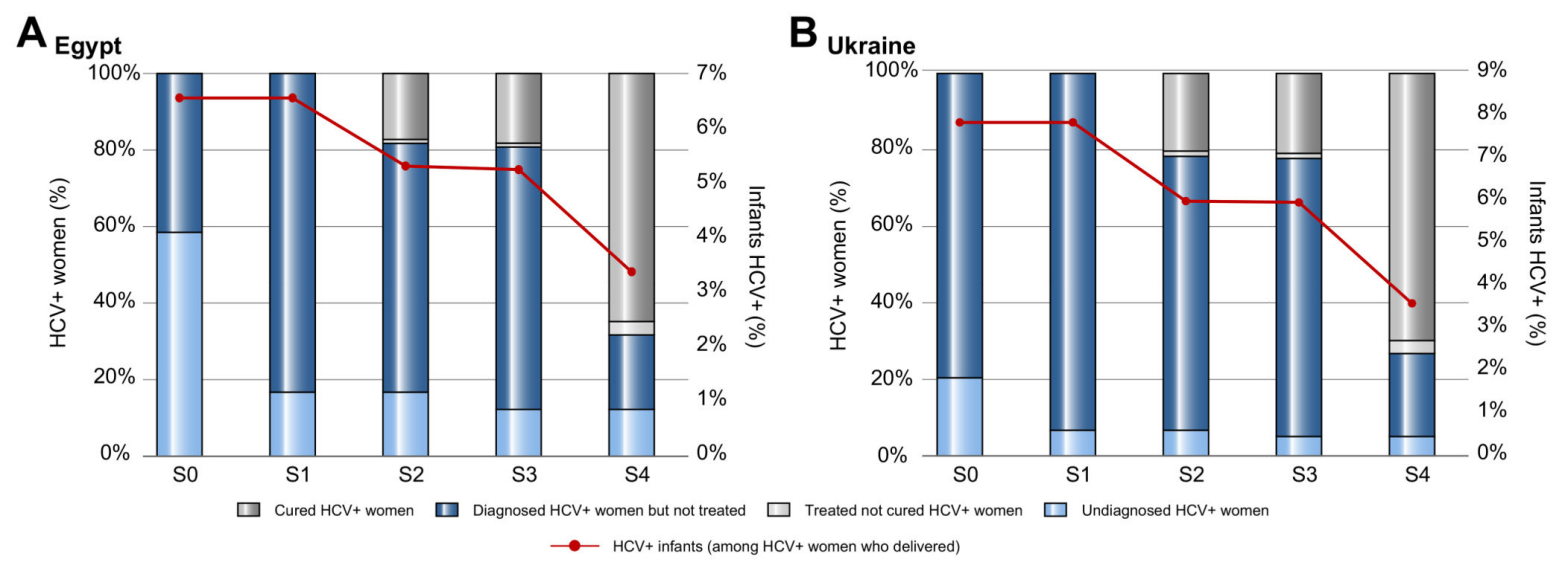
Baseline results for Egypt and Ukraine. (A) Egypt and (B) Ukraine. Proportions of HCV RNA positive (HCV+) women undiagnosed, diagnosed but untreated, diagnosed and cured by time of delivery or treated and not cured (left axis), and proportion of HCV+ infants at 6 months of age (right axis). The percentages for women are calculated among all HCV+ women who have started a pregnancy; the percentages of HCV+ infants at 6 months of age are calculated among HCV+ women who have given birth.

Among the 3 million pregnant women who have had at least one antenatal visit in Egypt, we estimated that 40,000 are HCV RNA positive, corresponding to a prevalence of 1.3%. When considering the SOC strategy (S0), with screening targeting pregnant women for whom a c-section is planned, 59% of women with HCV would remain undiagnosed by time of delivery (Table 4; Fig. 2A). Screening according to WHO recommendations (S1–S2) or universal screening (S3–S4) would reduce the proportion of undiagnosed women at delivery to 17% or 12%, respectively. Targeted DAA treatment for HCV RNA-positive women with VT risk factors would result in 17% or 18% of women being cured by delivery when combined with WHO recommended targeted screening (S2) or universal screening (S3) (Table 4; Fig. 2A). Universal screening and treatment (S4) would result in the highest proportion of women diagnosed and achieving HCV cure by delivery (65%), decreasing the number of HCV RNA-positive women at delivery to 14,100 (S4), *i.e*. 0.47% HCV RNA positive (-65% compared to S0) (supplementary information Fig. S1A, panel on the left).

In current SOC (*i.e*. deferring treatment to after delivery and cessation of breastfeeding [S0–S1]), 2,100 infants (6.5%) would be infected with HCV at 6 months of age, with this number decreasing to 1,700 (5.3%) with targeted DAA treatment during pregnancy (S1–S2) and 1,100 (3.4%) with universal treatment during pregnancy (S4) (Table 4; Fig. 2A). Compared to the SOC strategy (S0), this is a relative reduction of 19% and 48% in cases of VT (supplementary information Fig. S1A, panel on the right).

#### Ukraine

Among the 570,000 pregnant women who have had at least one antenatal visit in Ukraine, we estimated that 14,800 are HCV RNA positive *i.e*. 2.6% prevalence. Under the SOC strategy (S0) of targeted screening of only HIV-positive women, 20% of HCV RNA-positive women would remain undiagnosed by time of delivery (Table 4; Fig. 2B). Screening according to WHO recommendations (S1–S2) or universal screening (S3–S4) would reduce the proportion of undiagnosed women to 7% or 5%, respectively. Targeted DAA treatment for HCV RNA-positive women with VT risk factors would result in 20% to 21% of women being cured by time of delivery (S2–S3) (Table 4; Fig. 2B). Universal screening and treatment (S4) would result in the highest proportion of women diagnosed, achieving HCV cure by delivery in 70%, reducing the number of HCV RNA-positive women at delivery to 4,500 (S4), *i.e*. 0.78% HCV RNA positive (-70% compared to S0) (Fig. S1B, panel on the left).

In the deferred treatment strategies (S0–S1), 930 infants (7.9%) would be infected with HCV, this number decreases to 710 (6.0%) with targeted DAA treatment (S2–S3) and 420 (3.6%) with universal treatment (Table 4; Fig. 2B), this is a relative reduction of 24% and 55%, respectively, compared to the SOC strategy S0 (Fig. S1B, panel on the right).

### Sensitivity analysis

**Fig. 3:**
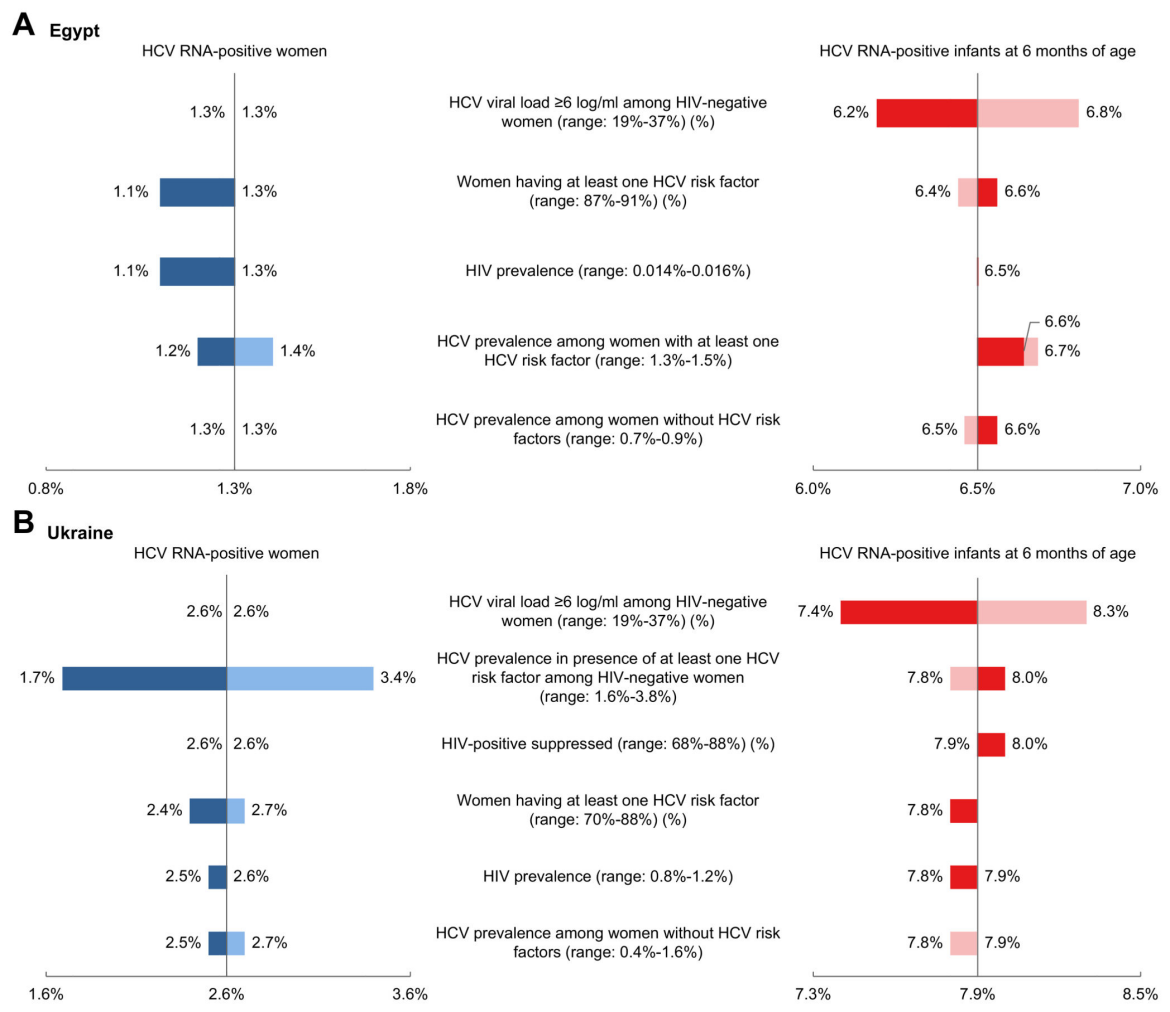
Univariate sensitivity analysis performed in both Egypt and Ukraine on the SOC strategy (S0). (A) Egypt and (B) Ukraine. S0, targeted risk-based screening (*i.e*. mainly, pregnant women with planned caesarean-section (c-section) in Egypt, pregnant women living with HIV in Ukraine) and no treatment during pregnancy (*i.e*. defer treatment to after pregnancy/breastfeeding). The tornado diagram summarises univariate sensitivity analysis to explore the robustness of the main outcomes (proportions of HCV RNA-positive women and HCV RNA-positive infants at 6 months of age) according to uncertainty of parameters. Each horizontal bar represents the range of the proportion of HCV RNA-positive women and HCV RNA-positive infants at 6 months of age calculated by varying the model parameters in a defined range.

Fig. 3A illustrates the results of sensitivity analysis for the SOC strategy (S0) for the parameters that had some impact on the proportions of HCV RNA-positive women at delivery and HCV RNA-positive infants at 6 months of age in Egypt. First, varying the proportion of women with HCV viral load ≥6log IU/ml among HIV-negative women (19%-37%) had the highest impact on the proportion of HCV RNA-positive infants at 6 months of age in S0 (variation by about 5%: 6.2%-6.8% compared to 6.5% in baseline analysis). The proportions of HCV RNA-positive women at the end of pregnancy did not change. Consequently, the magnitude of impact of alternative strategies compared to S0 was slightly higher or lower for VT estimates but did not change our conclusions (Fig. S2). For example, the proportion of HCV RNA-positive infants at 6 months of age with S4 would vary between 3.1% and 3.7% (compared to 3.4% in baseline), and the relative reduction in VT was 45-50% compared to SOC (*vs*. 48% in baseline) (Fig. S2B–2D). Other parameters had less impact on outcomes in S0 (Fig. 3A), leading to little or no change in the impact of alternative strategies compared to S0 (Figs. S3–S6).

Fig. 3B illustrates the results of similar sensitivity analyses applied to Ukraine. First, varying the proportion of women with HCV viral load ≥6log IU/ml among HIV-negative women (by about +/- 30%) again had the highest impact on the proportion of HCV RNA-positive infants (variation by about 6%: 7.4%-8.3% compared to 7.9% in baseline) (Fig. S7). As with Egypt, our conclusions remained the same. Second, varying the HCV prevalence in the presence of ≥1 HCV risk factor among HIV-negative women (1.6%-3.8%) had the greatest impact on the proportion of HCV RNA-positive women at delivery (variation by about 35%: 1.7%-3.4% compared to 2.6% in baseline). The impact on the proportion of HCV RNA-positive infants at 6 months of age was slightly lower (variation by about 1.3%: 7.8%-8.0% compared to 7.9% in baseline) and did not change the impacts of the alternative strategies compared to S0 (Fig. S8). Variation of other parameters had limited impact on our outcomes in S0 (Fig. 3B) or on the impact of alternative strategies compared to S0 (Figs. S9–S12).

**Fig. 4:**
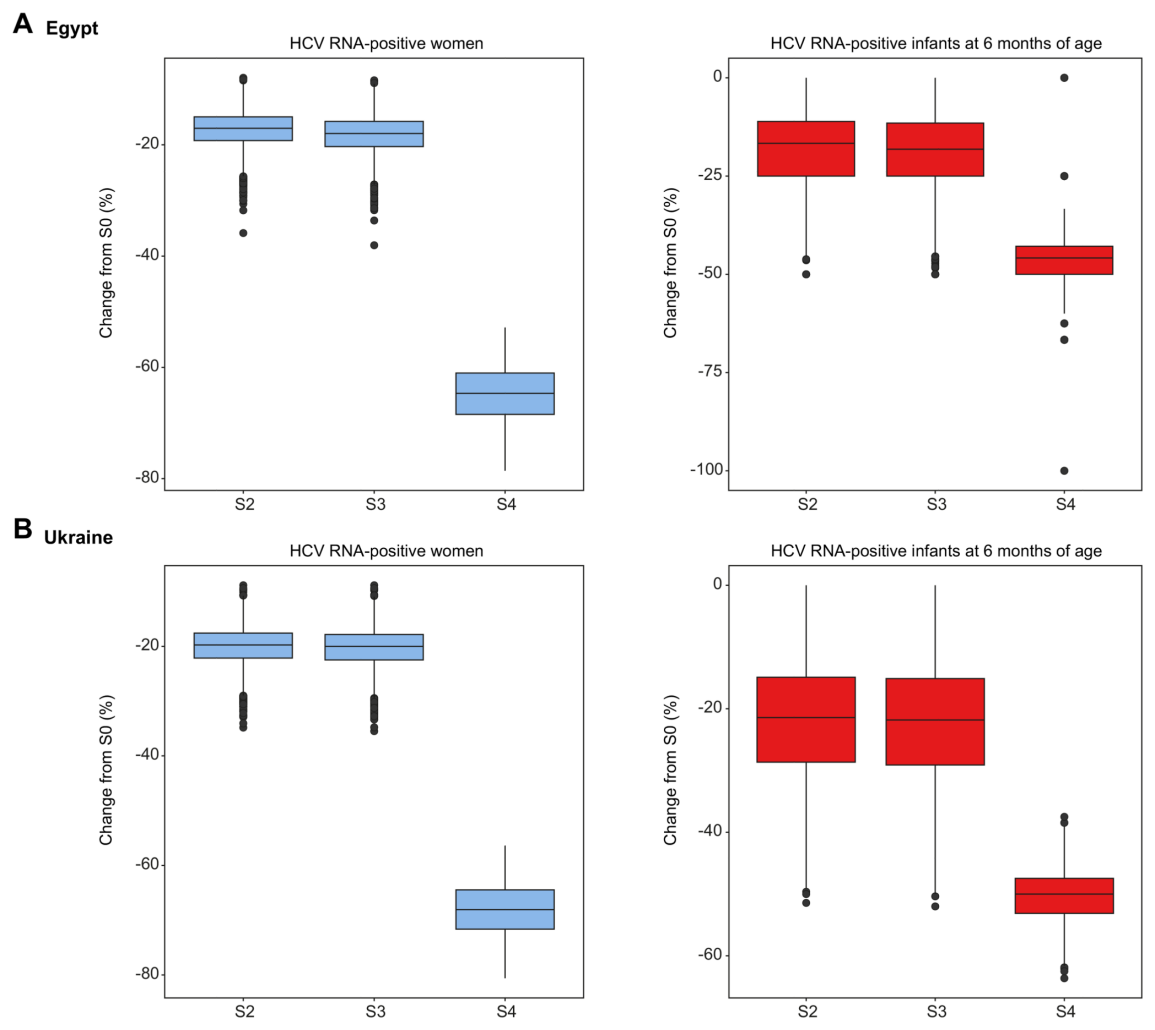
Boxplots of the relative change in proportions of HCV RNA-positive women and HCV-positive infants for the strategies S2 to S4 (S1 has no impact on these outcomes) compared to the SOC strategy (S0) in Egypt and Ukraine. (A) Egypt and (B) Ukraine. S0, targeted risk-based screening, no treatment during pregnancy; S2, WHO risk-based screening, targeted DAA during pregnancy; S3, Universal screening, targeted DAA during pregnancy; S4, Universal screening and DAA during pregnancy. The black line represents the median, the box represents the interquartile range (correspond to the distance between the first and third quartiles). Whiskers maximum distance is 1.5 times the interquartile range. Data beyond the end of the whiskers are called "outlying" points and are plotted individually. DAA, direct-acting antiviral; SOC, standard of care; WHO, World Health Organization.

Finally, simultaneously varying key parameters from appropriate probability distributions did not affect our overall findings. It provided some insights on the dispersion of the results for both contexts (Fig. 4).

## Discussion

This study is one of the first to explore the potential impact of different strategies for HCV screening and treatment of pregnant women in two countries with a high burden of HCV, Egypt and Ukraine. Assuming that DAAs are safe and efficacious for use in pregnancy and can help prevent VT, we found that universal screening and treatment of all pregnant women would result in the largest number of women being diagnosed during pregnancy and cured by delivery, with a significant decrease in the number of HCV RNA-positive infants at 6 months of age compared to all other alternative strategies of targeted/universal screening with deferred or targeted treatment. These findings were consistent when considering the range of uncertainties in our parameters in sensitivity analyses. The model assumes pregnant women initiate treatment from the third trimester based on the strategy of ongoing trials; if earlier treatment initiation was deemed safe then this could potentially increase maternal cure rates by time of delivery, which may further reduce the risk of VT.

We have shown that risk-based screening according to WHO recommendations (S1–S2) results in most mothers being aware of their infections at delivery (83% and 93%, respectively, in Egypt and Ukraine). These strategies, even if they do not include DAA treatment during pregnancy, would result in increased HCV diagnosis, which would enable referral for HCV treatment after delivery or the end of breastfeeding. High up-take of DAA treatment post-partum would additionally reduce the risk of VT for future pregnancies. It would also help identify HCV-exposed infants in need of early screening during the first months of life and it would enable prompt treatment initiation from 3 years in infants who are HCV RNA positive.^35^ A minor improvement in outcomes is observed by extending from targeted to universal screening in S3, the strategy S2 would likely be more attractive if there is no joint commitment to universal screening and universal treatment.

Increasing screening according to WHO recommendations combined with targeted treatment during pregnancy (S2) would cure 17% of women by delivery in Egypt and 20% in Ukraine, and decrease the proportion of HCV RNA-positive infants at 6 months of age to 5.3% and 6% (*i.e*. relative reduction by 19% and 24%, respectively, compared to deferred treatment in SOC). DAA treatment targeting women with risk factors for VT might be considered in settings where such risk factors are frequent, for example where HIV prevalence is high. This may be considered as an alternative to universal treatment, although a full cost-effectiveness analysis would be required. More data are needed to support the model assumptions and costing estimates to inform future research, policy and practice. Apart from hepatitis C, antenatal HIV treatment of pregnant women has been shown to improve both paediatric and maternal outcomes.^36^

Pregnancy provides an ideal opportunity to screen and treat women of childbearing age while they are engaged with healthcare providers, as it has the potential for dual dividends. First testing and treatment of pregnant women will cure them of chronic HCV, thereby averting the risk of disease progression and potentially reducing the risk of costly adverse maternal and infant outcomes associated with HCV status in pregnancy. Second are the benefits to her offspring, screening women will identify HCV-exposed infants in need of testing. Curing women during pregnancy will likely reduce the risk of VT for the current pregnancy and eliminate the risk for future pregnancies. There are also broader benefits in terms of decreasing HCV prevalence and risk of onward transmission in the community.

Future areas of research include a cost-effectiveness analysis of adding HCV screening (and treatment) to existing antenatal care screening policies across different settings. More broadly, it would be important to assess the added cost, benefit and feasibility of incorporating HCV into the WHO’s recommendation for triple elimination of HIV, HBV and syphilis through screening in pregnancy.^37^

Our work has some limitations. First, our study is based on a mathematical model which relied on certain assumptions when data were lacking. In particular, our study assumed that women living with HIV will all have their HIV status known once in ANC, and women who are newly diagnosed with HIV and on antiretroviral therapy will be virally suppressed by the time of starting HCV treatment. This assumption is more important in Ukraine where HIV prevalence is relatively high and may lead to an under-estimation of the risk of VT in this country. However, varying values of all input parameters in the sensitivity analysis did not significantly affect our results. Second, no published data were available regarding uptake of HCV screening during pregnancy in routine care. Our uptake estimates were based on findings from a survey on the acceptability of HCV screening in pregnant women in Ukraine which may not reflect uptake in practice.^20^ By contrast, for Egypt, some assumptions on uptake were based on expert opinion. Also, in both contexts, uptake of treatment during pregnancy was based on an acceptability survey where this was posed as a hypothetical question based on the scenario that DAAs were approved for use in pregnancy, again actual uptake is unknown although wide variations were explored in the sensitivity analysis. Finally, our model considered VT rates based on infant HCV RNA status at 6 months of age, which were based on modelled estimates from historical studies on VT.^8,34^ An alternative approach would be to use VT rates at time of delivery although this would ignore subsequent spontaneous clearance in early life and would not change our main findings.

In conclusion, this is one of the first models to demonstrate the potential benefits of increased HCV screening and treatment in pregnancy in two LMICs with generalised and concentrated HCV epidemics. There is increasing recognition that the elimination of HCV cannot leave entire subpopulations of pregnant women and young children behind.^9^ Future trials are needed to assess the safety and efficacy of DAA treatment in pregnancy, as well as its impact on VT, and findings from such trials will be critical in informing future models and cost-effectiveness analyses to guide future policy and practice.

## Supplementary Material

Supplemental information

## Figures and Tables

**Table 1:** Screening and treatment strategies evaluated in the decision model.

		Screening			Treatment
	Brief description	Egypt	Ukraine		All settings
S0	Targeted risk-based screening and deferred treatment	Mainly focused on women with planned caesarean-section (c-section)	Mainly focused on women living with HIV		Defer treatment to after delivery and cessation of breastfeeding
S1	WHO risk-based screening and deferred treatment	Risk-based screening (WHO recommendations)*			Defer treatment to after delivery and cessation of breastfeeding
S2	WHO risk-based screening and targeted DAA during pregnancy	Risk-based screening (WHO recommendations)*			DAA treatment during pregnancy for HCV RNA-positive women with ≥ 1 risk factor for HCV vertical transmission**
S3	Universal screening, targeted DAA during pregnancy	Universal screening of all pregnant women			DAA treatment during pregnancy for HCV RNA-positive women with ≥ 1 risk factor for HCV vertical transmission**
S4	Universal screening and DAA during pregnancy	Universal screening of all pregnant women			DAA treatment during pregnancy for all HCV RNA-positive women

DAA, direct-acting antiviral; WHO, World Health Organization.

* Based on HCV infection risk factors: People who have received medical or dental interventions in healthcare settings where infection control practices are substandard; people who have received blood transfusions prior to the time when serological testing of blood donors for HCV was initiated or in countries where serological testing of blood donations for HCV is not routinely performed; people who inject drugs; people who have had tattoos, body piercing or scarification procedures done where infection control practices are sub-standard; children born to mothers infected with HCV; people with HIV infection; people who use/have used intranasal drugs; prisoners and previously incarcerated persons.

** Risk factors for HCV vertical transmission: presence of HIV unsuppressed infection and/or high HCV viral load (≥6 log IU/ml).

**Table 2:** Main model parameter values for both countries.

	Egypt	Ukraine	References
Number of pregnant women in one year	3,000,000	570,000	^23–26^
HIV prevalence, % (range)	0.015 (0.014–0.016)	1.0 (0.8–1.2)	^27,28^
Viral suppression among HIV infection (viral load <1,000 copies/ml), % (range)	24 (22–26)	86 (68–88)	^27,28^
Presence of at least one HCV risk factor among HIV-negative, % (range)	89 (87–91)	79 (71–87)	^20,29^
Prevalence of HCV RNA in the presence of at least one HCV infection risk factor, % (range)			
HIV-positive	1.4 (1.3–1.5)	27 (25–29)	^29–31^
HIV-negative	1.4 (1.3–1.5)	2.7 (1.6–3.8)	^29,31^
Prevalence of HCV RNA in the absence of any HCV risk factor, % (range)	0.8 (0.7–0.9)	1 (0.4–1.6)	^29,31^
Prevalence of high HCV viral load (≥6 log IU/ml) among HCV RNA positive, % (range)			
HIV-positive	44 (30–59)	44 (30–59)	^7^
HIV-negative	28 (19–37)	28 (19–37)	
% of women attending ANC at			^32,33^
3 months	77.9	85.3	
5 months	89.7	97.1	
7 months	92.5	99.0	
8 months	93.4	99.3	
9 months	100	100	

**Table 3:** Data and assumptions on the uptake of HCV screening and treatment in both settings, for each strategy.

	S0	S1	S2	S3	S4
**Egypt**					
Uptake of screening					
Absence of HCV RF*	30% when planned c-section, 10% otherwise			88%	88%
At least one HCV RF*	60% when planned c-section, 30% otherwise	88%	88%	88%	88%
Uptake of treatment ^†^	0%	0%	78% of women with VT RF^‡^		78% of all HCV+ women
**Ukraine**					
Uptake of screening					
Absence of HCV RF*	75%	75%	75%	95%	95%
At least one HCV RF*	87% when HIV, 79% otherwise	95%	95%	95%	95%
Uptake of treatment^†^	0%	0%	78% of women with VT RF^‡^		78% of all HCV+ women

RF, risk factor; VT, vertical transmission.

S0, targeted risk-based screening, no treatment during pregnancy; S1, WHO risk-based screening, no treatment during pregnancy; S2, WHO risk-based screening, targeted DAA during pregnancy; S3, Universal screening, targeted DAA during pregnancy; S4, Universal screening and DAA during pregnancy.

* HCV RF, HCV risk factor (2): Persons who have received medical or dental interventions in healthcare settings where infection control practices are substandard, Persons who have received blood transfusions prior to the time when serological testing of blood donors for HCV was started or in countries where serological testing of blood donations for HCV is not routinely performed, People who inject drugs (PWID), Persons who have had tattoos, body piercing or scarification procedures done where infection control practices are sub-standard, Children born to mothers living with HCV, Persons with HIV infection, Persons who use/have used intranasal drugs, Prisoners and previously incarcerated persons.

† Treatment can be started from the third trimester month of pregnancy.

‡ VT RF, vertical transmission risk factors when HCV RNA positive: presence of HIV infection and/or high HCV viral load (≥6 log IU/ml).

**Table 4:** Baseline effectiveness analysis in both settings, for each strategy.

	S0	S1	S2	S3	S4
**Egypt** *					
Women remaining HCV RNA positive at delivery	100%**	100%	83%	82%	35%
Undiagnosed HCV RNA-positive pregnant women	59%	17%	17%	12%	12%
Diagnosed HCV RNA-positive pregnant women but not treated	42%	83%	65%	69%	20%
Cured women after DAA treatment	–	–	17%	18%	65%
HCV RNA-positive infants at 6 months of age	6.5%	6.5%	5.3%	5.3%	3.4%
**Ukraine** *					
Women remaining HCV RNA positive at delivery	100%**	100%	80%	79%	30%
Undiagnosed HCV RNA-positive pregnant women	20%	7%	7%	5%	5%
Diagnosed HCV RNA-positive pregnant women but not treated	79%	93%	72%	73%	22%
Cured women after DAA treatment	–	–	20%	21%	70%
HCV RNA-positive infants at 6 months of age	7.9%	7.9%	6.0%	6.0%	3.6%

DAA, direct-acting antiviral; WHO, World Health Organization.

S0, targeted risk-based screening, no treatment during pregnancy; S1, WHO risk-based screening, no treatment during pregnancy; S2, WHO risk-based screening, targeted DAA during pregnancy; S3, Universal screening, targeted DAA during pregnancy; S4, Universal screening and DAA during pregnancy.

* For women, proportions are calculated among all HCV RNA positive women who have started a pregnancy; for HCV RNA-positive infants, they are calculated among HCV RNA positive women who have given birth.

** There are 40,000 and 14,800 HCV RNA positive women in Egypt and Ukraine respectively.

## Data Availability

Most of the data that support the findings of this study are available from the cited references and supplementary information. Some data regarding the acceptability of screening and treatment were calculated ad-hoc by Karen Scott, one of the co-authors, basing on the acceptability survey of Hepatitis C screening and treatment during pregnancy in pregnant women in Egypt, Pakistan and Ukraine (reference 20 in this work). The data concerning the level of HCV viral load in HCV-positive women according to their HIV serological status were calculated ad-hoc by Eugénia Mariné-Barjoan, one of the co-authors, owner of the database which allowed publication on risk factors for mother-to-child transmission of hepatitis C virus (reference 7 in this work). Other data are available from the authors upon reasonable request.

## Abbreviations

ANC: antenatal care
DAAs: direct-acting antivirals
LMICs: lower middle-income countries
SOC: standard of care
VT: vertical transmission
WHO: World Health Organization.
