# Supplementary material for: Modelling the potential effectiveness of hepatitis C screening and treatment strategies during pregnancy in Egypt and Ukraine
Source: J Hepatol. Author manuscript; Available in PMC 2024 Aug 8. (PMC7616347; doi:10.1016/j.jhep.2022.12.032)
Supplement: Supplemental information [file EMS197914-supplement-Supplemental_information.pdf]

Journal of Hepatology, Volume ■

Supplemental information

Modelling the potential effectiveness of hepatitis C screening and treatment strategies during pregnancy in Egypt and Ukraine

Nadia Hachicha-Maalej, Intira Jeannie Collins, Anthony E. Ades, Karen Scott, Ali Judd, Aya Mostafa, Elizabeth Chappell, Manal Hamdy-El-Sayed, Diana Gibb, Sarah Pett, Eugènia Mariné-Barjoan, Alla Volokha, Yazdan Yazdanpanah, and Sylvie Deuffic-Burban

Modelling the potential effectiveness of hepatitis C screening and treatment strategies during pregnancy in Egypt and Ukraine

Nadia Hachicha Maalej, Intira Jeannie Collins, AE Ades, Karen Scott, Ali Judd, Aya Mostafa, Elizabeth Chappell, Manal Hamdy-El-Sayed, Diana Gibb, Sarah Pett, Eugènia Mariné-Barjoan, Alla Volokha, Yazdan Yazdanpanah, Sylvie Deuffic-Burban

Table of contents

Input parameters 3

Study population 3

Vertical transmission 5

Sensitivity analysis 6

Fig. S1. 7

Fig. S2 8

Fig. S3 9

Fig. S4 10

Fig. S5 11

Fig. S6. 12

Fig. S7 13

Fig. S8 14

Fig. S9 15

Fig. S10. 16

Fig. S11 17

Fig. S12. 18

Table S1 19

Table S2 20

Table S3 23

Supplementary references.. 32

Input parameters

Study population

Number of pregnancies: For the Egyptian context, based on the annual number of births (1), we reconstructed the number of women who started a pregnancy (with or without a birth), having had at least one antenatal visit (2). We took into account fetal loss rate applied in the model (3) and twinning rate (4) to obtain about 3 million pregnancies. For the Ukrainian context, a number of 860,000 pregnancies each year is estimated by Stover et al. (5). We deduced the voluntary terminations of pregnancy occurring during the first 8 weeks estimated by this study, assuming that most women who interrupt their pregnancy before 8 weeks will not necessarily be followed up in antenatal care. We also deduced non-followed-up pregnancies (6) to obtain 570,000 pregnancies.

HIV suppression: We assumed that HIV status is known at access to ANC. Among HIV-positive women, a proportion received ARV treatment for prevention of HIV mother-to-child transmission during ANC in Egypt and Ukraine, respectively 27% and 95% (7,8). We assumed that 90% of them would rapidly achieve HIV viral suppression (i.e. HIV viral load <1000 copies/mL) (9,10). We assumed the same risk of HCV VT among women with HIV suppressed coinfection HCV mono-infected women (11).

Prevalence of HCV risk factors and HCV RNA: In the context of Egypt, data on the presence of at least one HCV risk factor were derived from a cross-sectional single centre study among pregnant women attending an antenatal clinic in a tertiary care facility (12). HIV infection is rare in Egypt, and we considered that HCV prevalence was not dependent on women's HIV status. We obtained the prevalence of HCV RNA according to the presence or absence of HCV risk factors from the overall HCV RNA prevalence among women of childbearing age (13) (1.38% of women aged 15-49 years were HCV RNA+), assuming that 89% of women had at least one risk factor for HCV infection and the odds ratio of being HCV-infected according to

the presence or absence of risk factors; a woman with at least one HCV risk factor being 1.77 times more likely to be HCV RNA+ than a woman without any risk factor. Thus, the prevalence of HCV RNA was estimated at 1.4% in the presence of at least one HCV risk factor, and 0.8% otherwise. In the context of Ukraine, the prevalence of HCV RNA was defined for HIV-positive women, and according to the presence or absence of HCV risk factors for HIV-negative women. It was estimated at 27% among women living with HIV from the estimated 33% of anti-HCV positive women of which 82% were HCV RNA positive (14). It was estimated that 79% of HIV-negative women had at least one HCV risk factor. We used data among blood donors as a proxy of HCV prevalence in the absence of risk factors (anti-HCV prevalence of 1.3%), and calculated HCV prevalence for pregnant women having at least one risk factor other than HIV assuming a 2.7% anti-HCV prevalence in the general population. Assuming a 74% prevalence of HCV RNA among antibody-HCV positive women, we obtained a prevalence of HCV RNA of 2.7% in the presence of at least one HCV risk factor, and 1% in the absence of HCV risk factors (15).

Mode of delivery: Mode of delivery is a selection criterion in the screening strategy implemented in Egypt. It also enters into the calculation of the probability of transmission. The rate of deliveries by c-section was estimated at 44% for Egypt's context and to 12% for Ukraine's context (16,17).

Duration of pregnancies: due to lack of local data, we used French published data to obtain a monthly distribution of pregnancy duration (18). Given the relatively high prevalence of HIV in Ukraine and a reported higher risk of preterm birth in HIV-positive pregnant women, we adjusted the monthly probabilities of deliveries in this subgroup (19).

Mortality: In both settings, maternal mortality was applied using WHO estimations (1,20); risk of fetal loss, i.e. a miscarriage occurring during the first 6 months of pregnancy, and stillbirth

occurring during the 3rd trimester of pregnancy, were included and derived from local studies taking into account a higher risk of fetal loss in HCV-RNA positive women in Egypt (3,5).

Vertical transmission

Estimates of vertical transmission probabilities were obtained from a Bayesian multi-parameter evidence synthesis from European data on individual mother-child pairs (21). This analysis estimates the risk of vertical infection, the impact of risk factors (mother's HIV and HCV RNA viral load), and the proportions of infection transmitted Early in Utero (EiU), Late in Utero (LiU) and at delivery.

Let $V_{EiU,g}$, $V_{LiU,g}$ and $V_{Del,g}$ be the probabilities of transmission for infants in group g at each stage conditional on the absence of transmission at a previous stage, where g represents the risk group (combination of HIV-infection/ HIV-non infection, HCV high viral load/ HCV low viral load and mode of delivery). Probabilities of "ever" infection π are defined in infants delivered by elective caesarean (ECS) and in those delivered by other mode of delivery (non-ECS):

$$\pi_g = \begin{cases} V_{EiU,g} + (1 - V_{EiU,g})V_{LiU,g} & ECS \\ V_{EiU,g} + (1 - V_{EiU,g})V_{LiU,g} + (1 - V_{EiU,g})(1 - V_{LiU,g})V_{Del,g} & non - ECS \end{cases}$$

Taking g=1 as representing HIV-negative and low viral load group, the risk of transmission in other groups is:

$$logit(V_{EiU,g}) = logit(V_{EiU,1}) + \delta_h H_g + \delta_v V_g + \delta_x X_g$$

$$logit(V_{LiU,g}) = logit(V_{LiU,1}) + \delta_h H_g + \delta_v V_g + \delta_x X_g$$

$$logit(V_{Del,g}) = logit(V_{Del,1}) + \delta_h H_g + \delta_v V_g + \delta_x X_g$$

Where:

$V_{EiU,1}$, $V_{LiU,1}$ and $V_{Del,1}$are stage-specific transmission probabilities in the low-risk group (g=1) δ_h, δ_v and δ_x are log odds ratios for HIV-positive relative to HIV-negative, high viral load relative to low viral load and interaction HIV-positive and high viral load relative to HIV-negative and low viral load. Similarly, H_g, V_g and X_g are indicators for the cited risk factors.

The estimated transmissions using the latter equations reflected overall (ever occurred) transmissions. In their analysis, Ades et al. estimated the overall VT rates at 7.2% in HIV-negative women and 12.1% in HIV-co-infected women (22). These overall VT rates ranged between 5.6% for low HCV viral load and delivery by ECS and 15.3% for high HCV viral load and delivery by non-ECS in HIV-negative women, and between 9.6% and 19.1%, respectively, in HIV-co-infected women. They also estimated clearance rates at different ages of the infant (22) that allow us to apply transmission net of clearance at 6 months.

Sensitivity analysis

When we varied simultaneously model parameter values, each value was replaced with the appropriate distribution according to ISPOR-SMDM Modelling Good Research Practices Task Force-6 (23). Parameters of the beta distribution were estimated from average values ($E(x)$) and standard errors (σ) – if known, 10% of the average otherwise – using the following equations:

$$\alpha = \left(\frac{E(x)^2/(1 - E(x)}{\sigma^2}\right) - E(x)$$

$$\beta = \frac{\alpha\big(1 - E(x)\big)}{E(x)}$$

Fig. S1. Relative change in proportions of HCV-RNA positive women at the end of pregnancy and HCV-positive infants for the strategies S1 to S4 compared to the SOC scenario (S0) in both contexts: A/ Egypt, B/ Ukraine.

S0, targeted risk-based screening, no treatment during pregnancy; S2, WHO risk-based screening, targeted DAA during pregnancy; S3, Universal screening, targeted DAA during pregnancy; S4, Universal screening and DAA during pregnancy.

A) Egypt

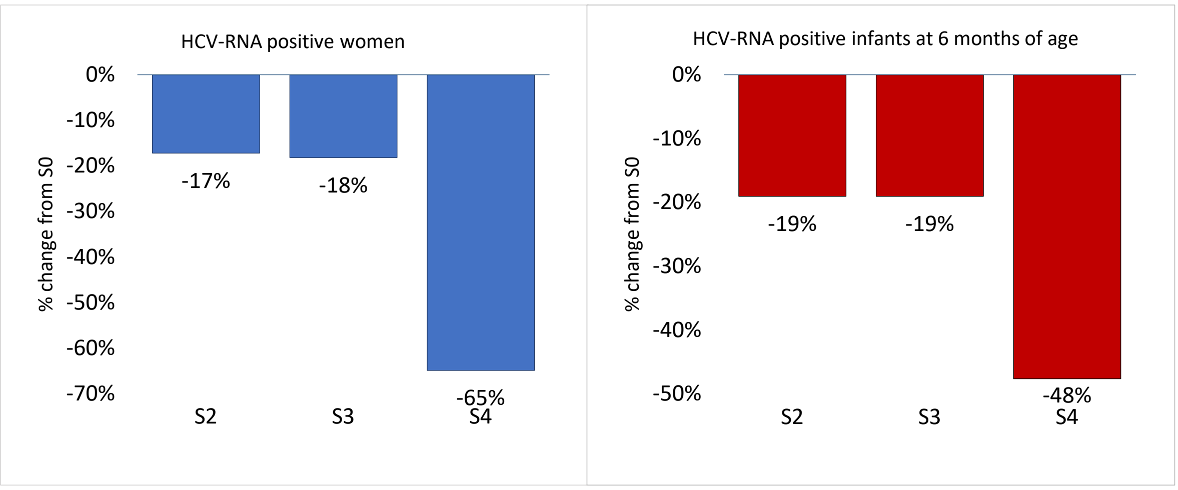

B) Ukraine

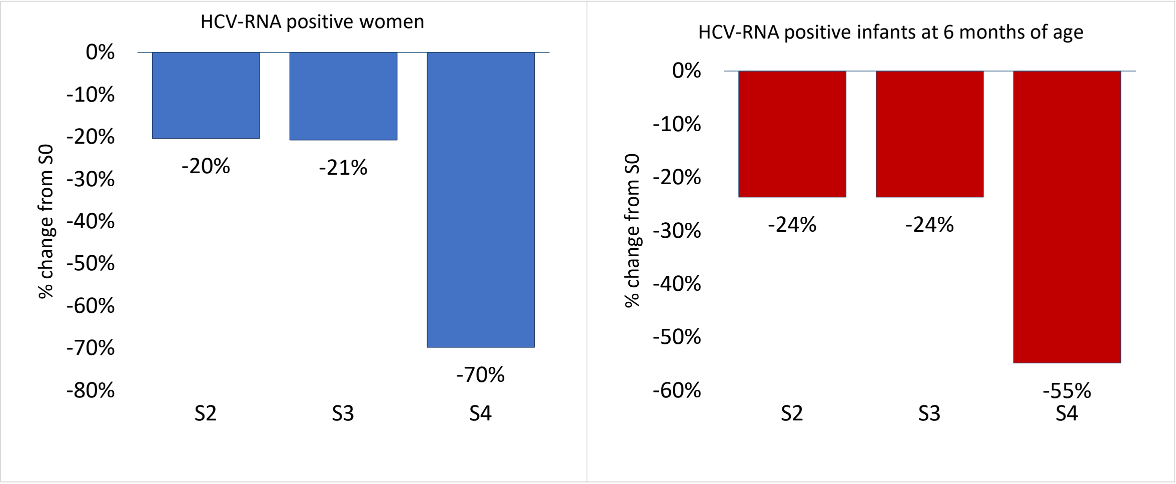

Fig. S2. Sensitivity analysis varying the proportion of women with HCV viral load ≥ 6log/mL among HIV-negative women in Egypt at 19% (A, B) and 37% (C, D) for the strategies S2 to S4 (S1 has no impact on both outcomes) compared to SOC strategy (S0). Relative change in proportions of HCV-RNA positive women at the end of pregnancy and in HCV-RNA positive infants at 6 months of age. S0, targeted risk-based screening, no treatment during pregnancy; S2, WHO risk-based screening, targeted DAA during pregnancy; S3, Universal screening, targeted DAA during pregnancy; S4, Universal screening and DAA during pregnancy.

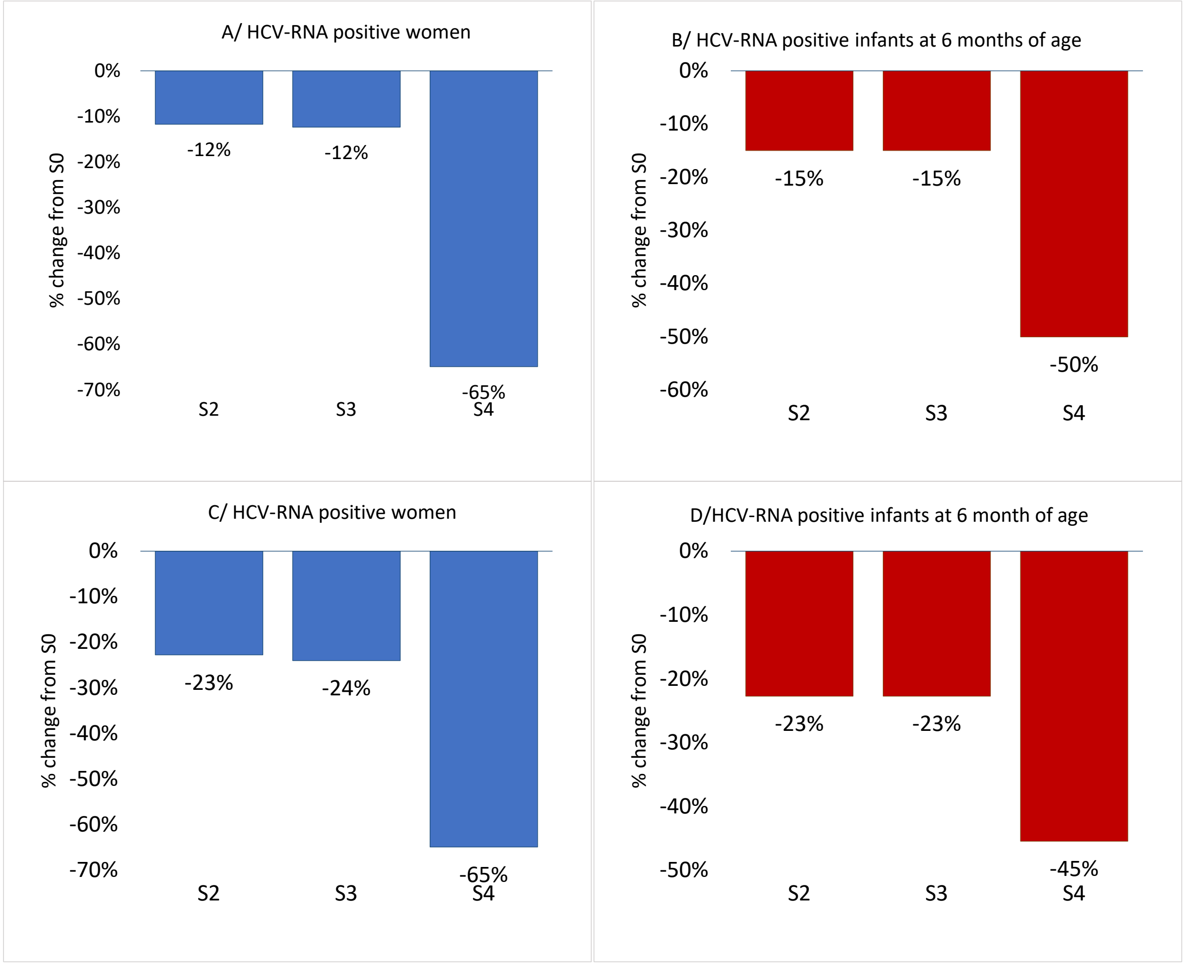

Fig. S3. Sensitivity analysis varying the proportion of women having at least one HCV risk factor in Egypt at 87% (A, B) and 91% (C, D) for the strategies S2 to S4 (S1 has no impact on both outcomes) compared to SOC strategy (S0).

Relative change in proportions of HCV-RNA positive women at the end of pregnancy and in HCV-RNA positive infants at 6 months of age. S0, targeted risk-based screening, no treatment during pregnancy; S2, WHO risk-based screening, targeted DAA during pregnancy; S3, Universal screening, targeted DAA during pregnancy; S4, Universal screening and DAA during pregnancy.

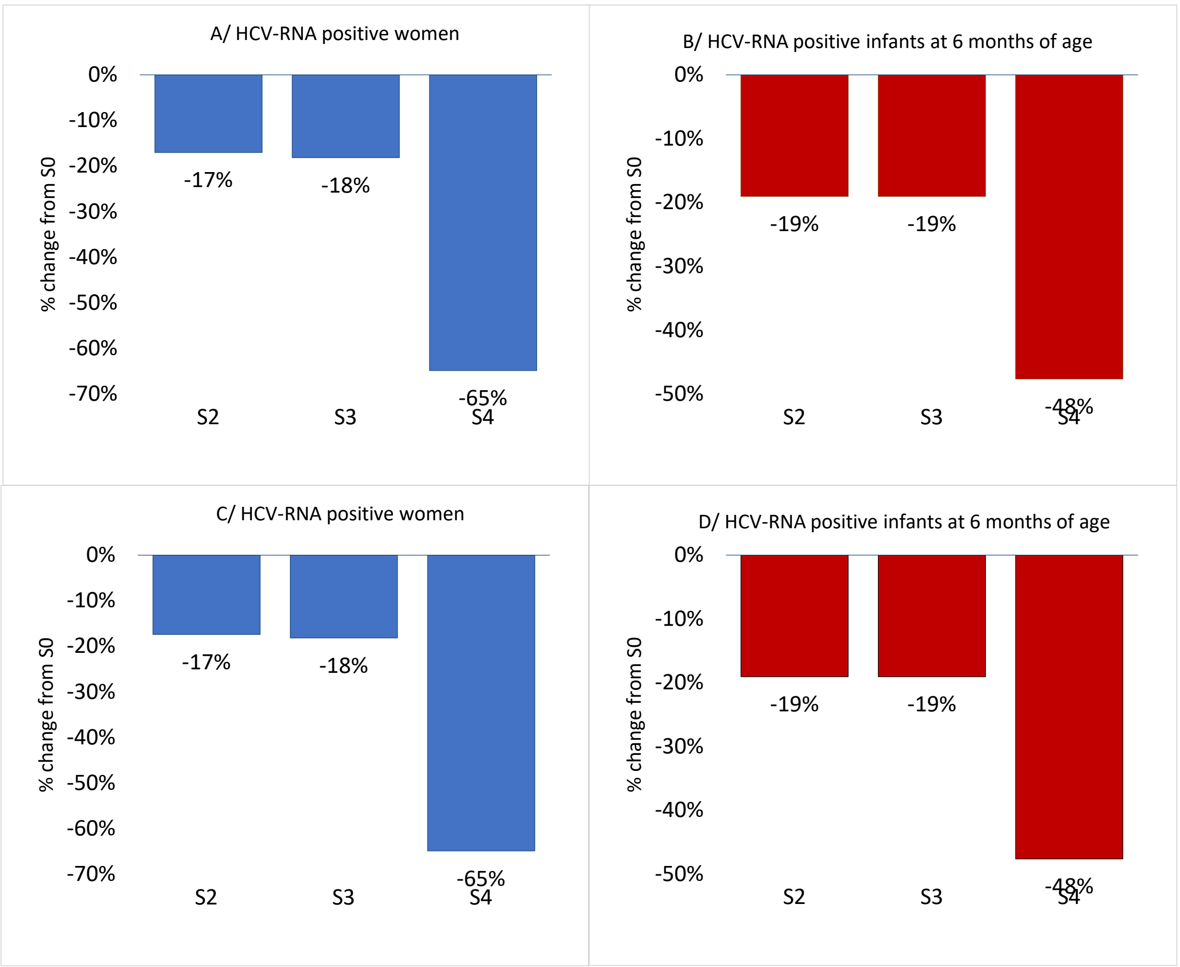

Fig. S4. Sensitivity analysis varying the HIV prevalence in Egypt at 0.014% (A, B) and 0.016% (C, D) for the strategies S2 to S4 (S1 has no impact on both outcomes) compared to SOC strategy (S0).

Relative change in proportions of HCV-RNA positive women at the end of pregnancy and in HCV-RNA positive infants at 6 months of age. S0, targeted risk-based screening, no treatment during pregnancy; S2, WHO risk-based screening, targeted DAA during pregnancy; S3, Universal screening, targeted DAA during pregnancy; S4, Universal screening and DAA during pregnancy.

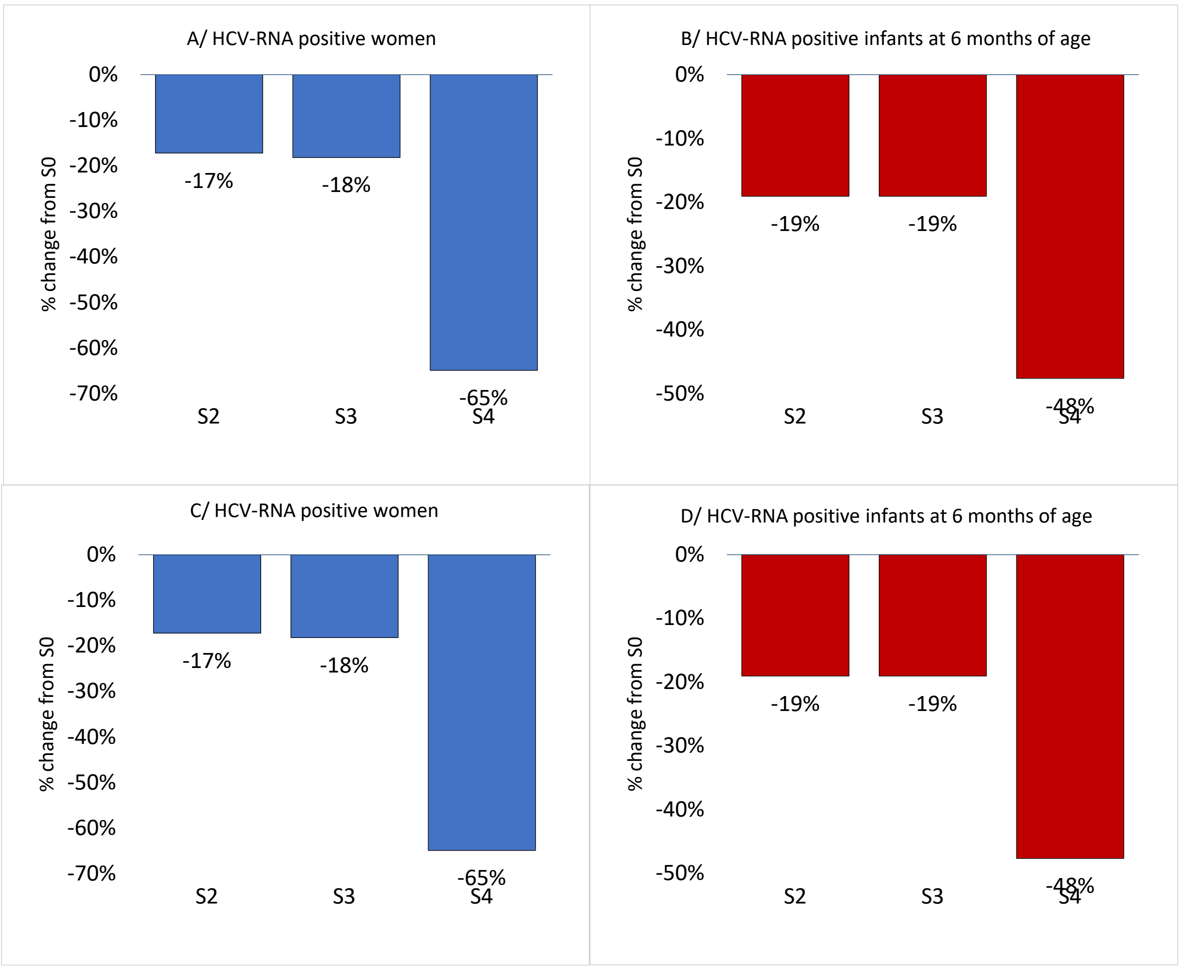

Fig. S5. Sensitivity analysis varying the HCV prevalence among women with at least one HCV risk factor in Egypt at 1.3% (A, B) and 1.5% (C, D) for the strategies S2 to S4 (S1 has no impact on both outcomes) compared to SOC strategy (S0).

Relative change in proportions of HCV-RNA positive women at the end of pregnancy and in HCV-RNA positive infants at 6 months of age. S0, targeted risk-based screening, no treatment during pregnancy; S2, WHO risk-based screening, targeted DAA during pregnancy; S3, Universal screening, targeted DAA during pregnancy; S4, Universal screening and DAA during pregnancy.

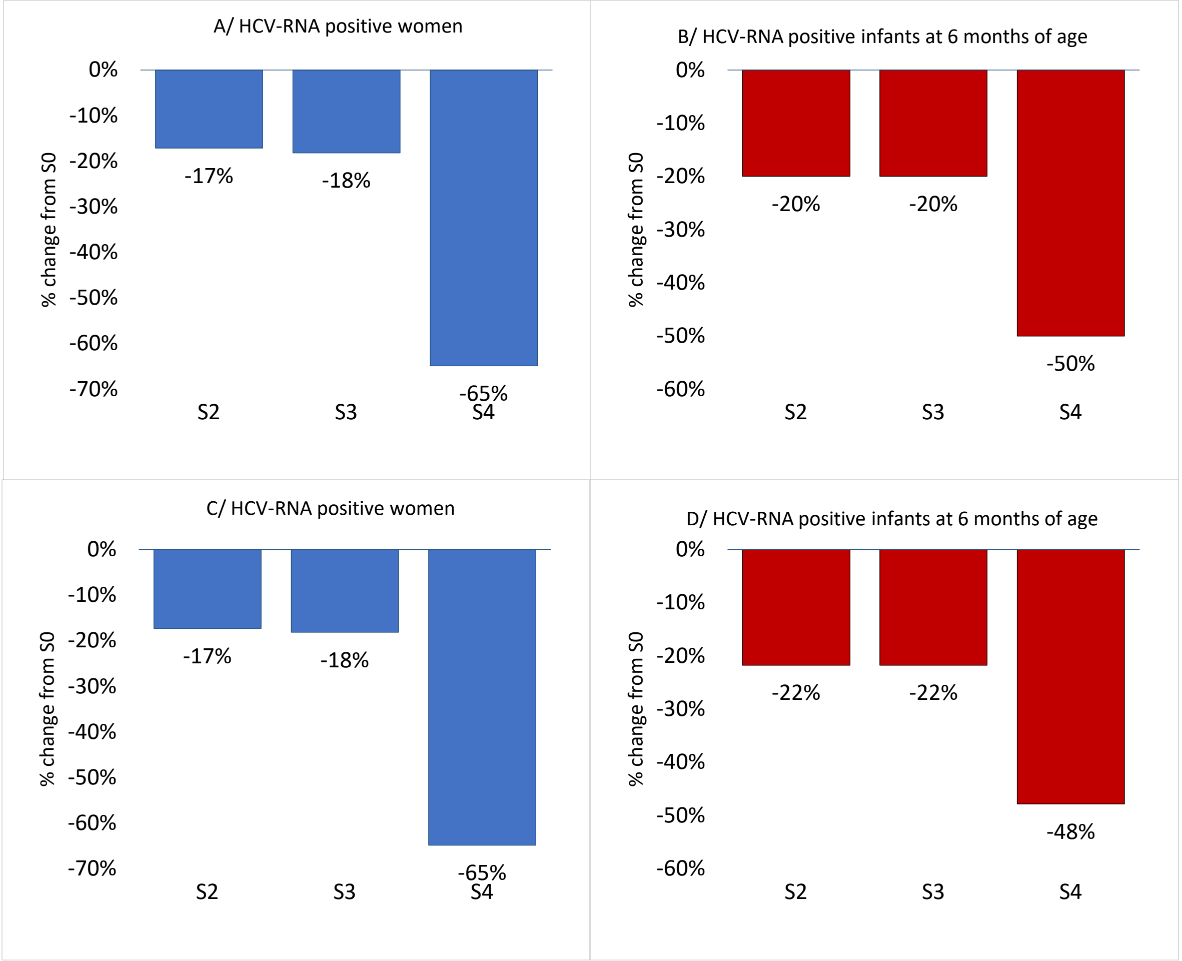

Fig. S6. Sensitivity analysis varying the HCV prevalence among women without HCV risk factors in Egypt at 0.7% (A, B) and 0.9% (C, D) for the strategies S2 to S4 (S1 has no impact on both outcomes) compared to SOC strategy (S0).

Relative change in proportions of HCV-RNA positive women at the end of pregnancy and in HCV-RNA positive infants at 6 months of age. S0, targeted risk-based screening, no treatment during pregnancy; S2, WHO risk-based screening, targeted DAA during pregnancy; S3, Universal screening, targeted DAA during pregnancy; S4, Universal screening and DAA during pregnancy.

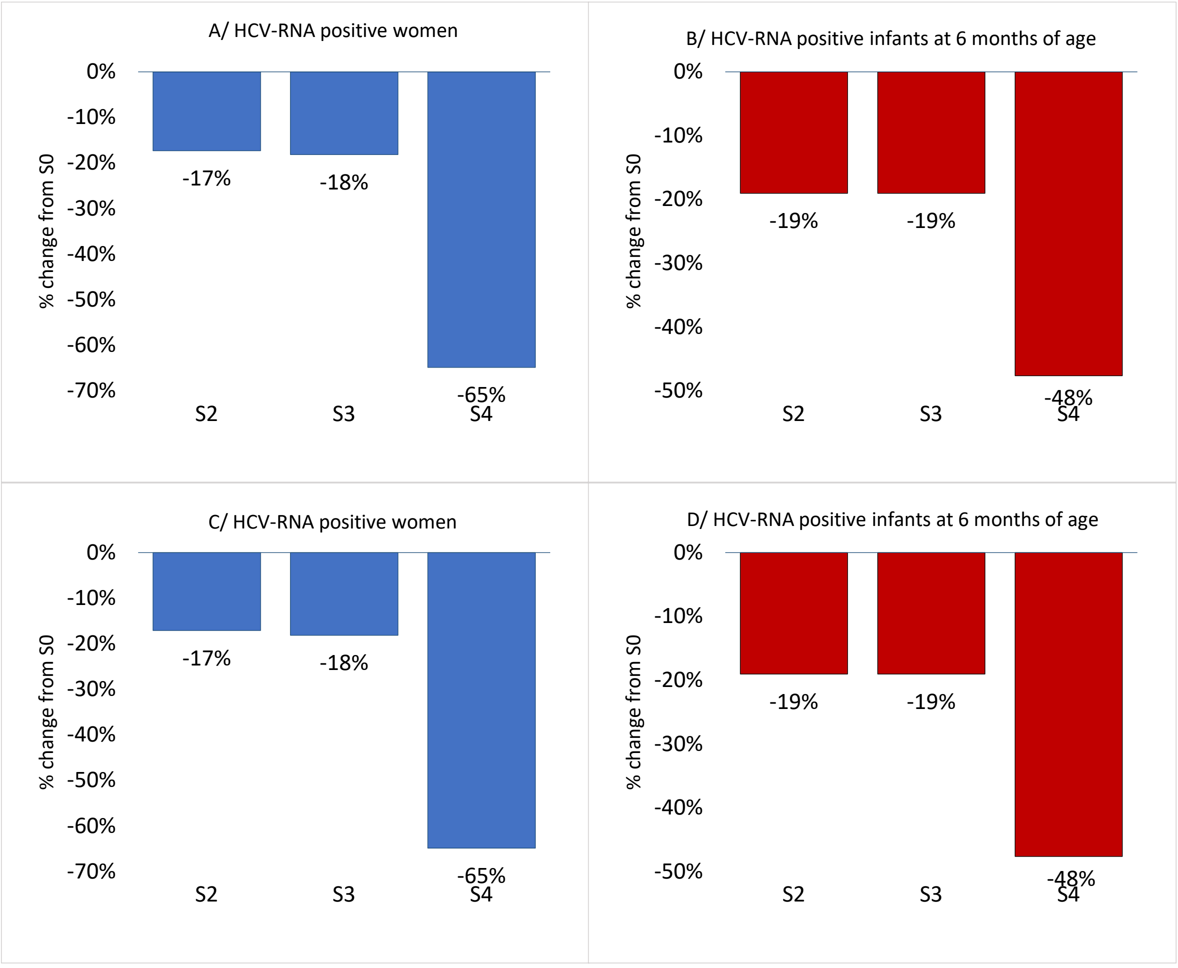

Fig. S7. Sensitivity analysis varying the proportion of women with HCV viral load ≥ 6log/mL among HIV-negative women in Ukraine at 19% (A, B) and 37% (C, D) for the strategies S2 to S4 (S1 has no impact on both outcomes) compared to SOC strategy (S0). Relative change in proportions of HCV-RNA positive women at the end of pregnancy and in HCV-RNA positive infants at 6 months of age. S0, targeted risk-based screening, no treatment during pregnancy; S2, WHO risk-based screening, targeted DAA during pregnancy; S3, Universal screening, targeted DAA during pregnancy; S4, Universal screening and DAA during pregnancy.

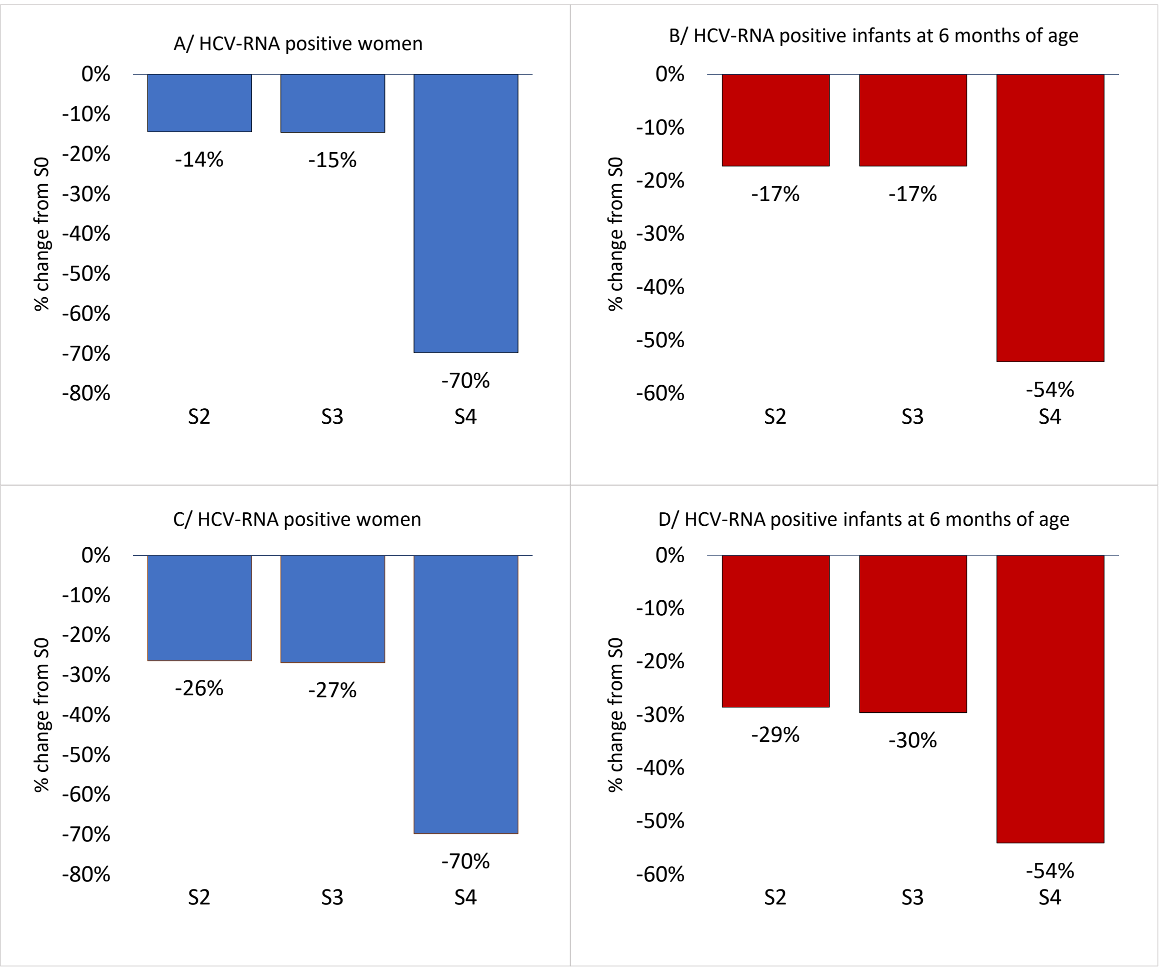

Fig. S8. Sensitivity analysis varying the HCV prevalence in the presence of at least one HCV risk factor among HIV-negative women in Ukraine at 1.6% (A, B) and 3.8% (C, D) for the strategies S2 to S4 (S1 has no impact on both outcomes) compared to SOC strategy (S0).

Relative change in proportions of HCV-RNA positive women at the end of pregnancy and in HCV-RNA positive infants at 6 months of age. S0, targeted risk-based screening, no treatment during pregnancy; S2, WHO risk-based screening, targeted DAA during pregnancy; S3, Universal screening, targeted DAA during pregnancy; S4, Universal screening and DAA during pregnancy.

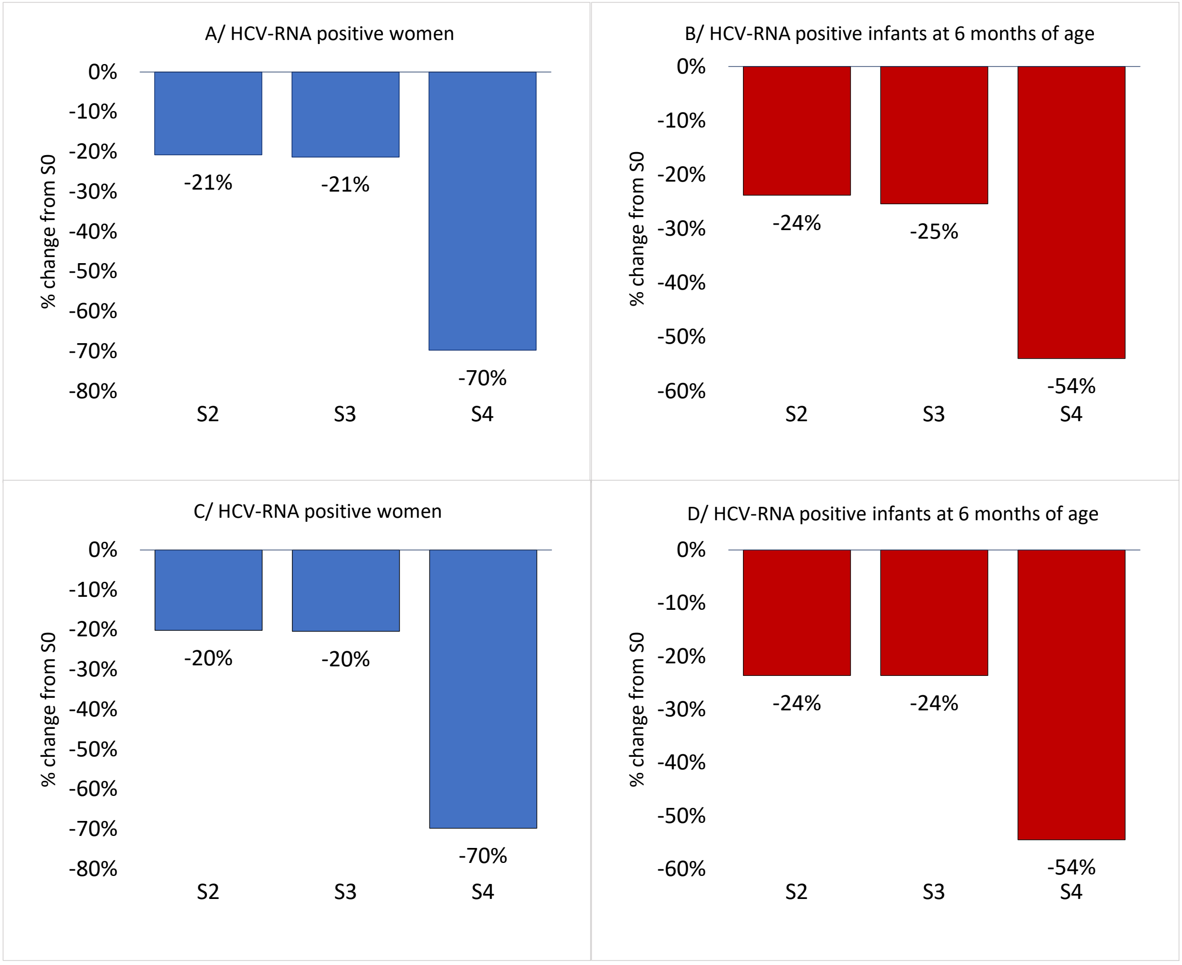

Fig. S9. Sensitivity analysis varying the proportion of HIV-positive suppressed women in Ukraine at 68% (A, B) and 88% (C, D) for the strategies S2 to S4 (S1 has no impact on both outcomes) compared to SOC strategy (S0).

Relative change in proportions of HCV-RNA positive women at the end of pregnancy and in HCV-RNA positive infants at 6 months of age. S0, targeted risk-based screening, no treatment during pregnancy; S2, WHO risk-based screening, targeted DAA during pregnancy; S3, Universal screening, targeted DAA during pregnancy; S4, Universal screening and DAA during pregnancy.

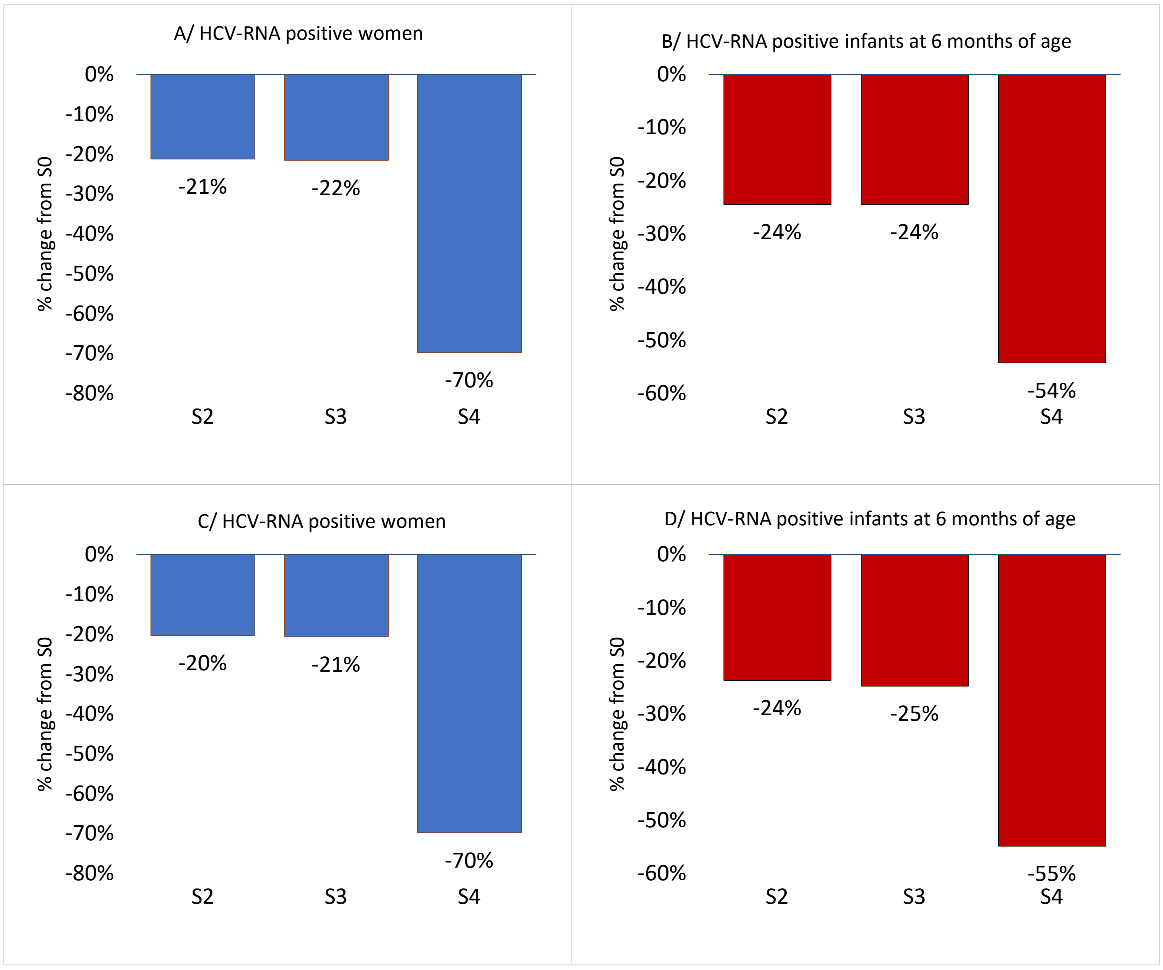

Fig. S10. Sensitivity analysis varying the proportion of women having at least one HCV risk factor in Ukraine at 70% (A, B) and 88% (C, D) for the strategies S2 to S4 (S1 has no impact on both outcomes) compared to SOC strategy (S0).

Relative change in proportions of HCV-RNA positive women at the end of pregnancy and in HCV-RNA positive infants at 6 months of age. S0, targeted risk-based screening, no treatment during pregnancy; S2, WHO risk-based screening, targeted DAA during pregnancy; S3, Universal screening, targeted DAA during pregnancy; S4, Universal screening and DAA during pregnancy.

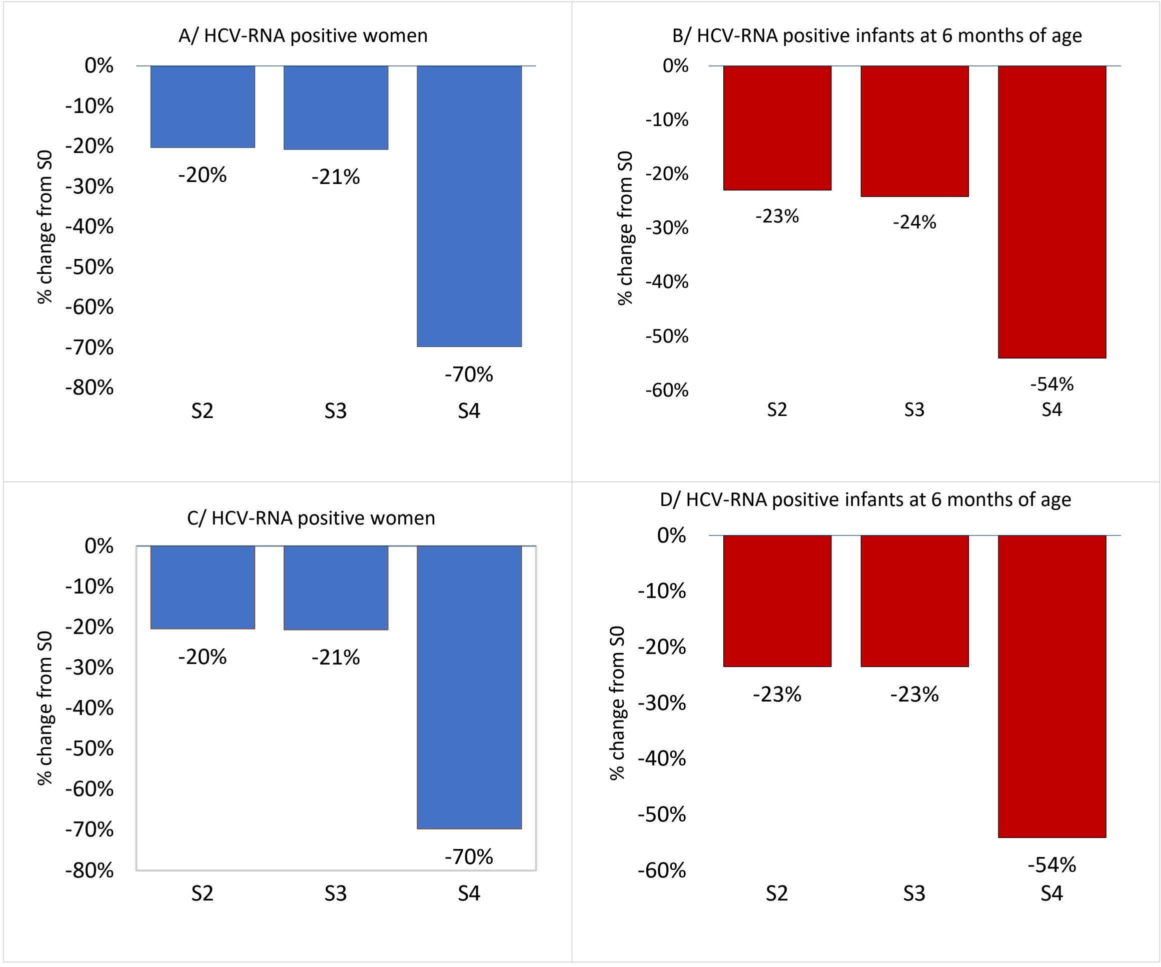

Fig. S11. Sensitivity analysis varying the HIV prevalence in Ukraine at 0.8% (A, B) and 1.2% (C, D) for the strategies S2 to S4 (S1 has no impact on both outcomes) compared to SOC strategy (S0).

Relative change in proportions of HCV-RNA positive women at the end of pregnancy and in HCV-RNA positive infants at 6 months of age. S0, targeted risk-based screening, no treatment during pregnancy; S2, WHO risk-based screening, targeted DAA during pregnancy; S3, Universal screening, targeted DAA during pregnancy; S4, Universal screening and DAA during pregnancy.

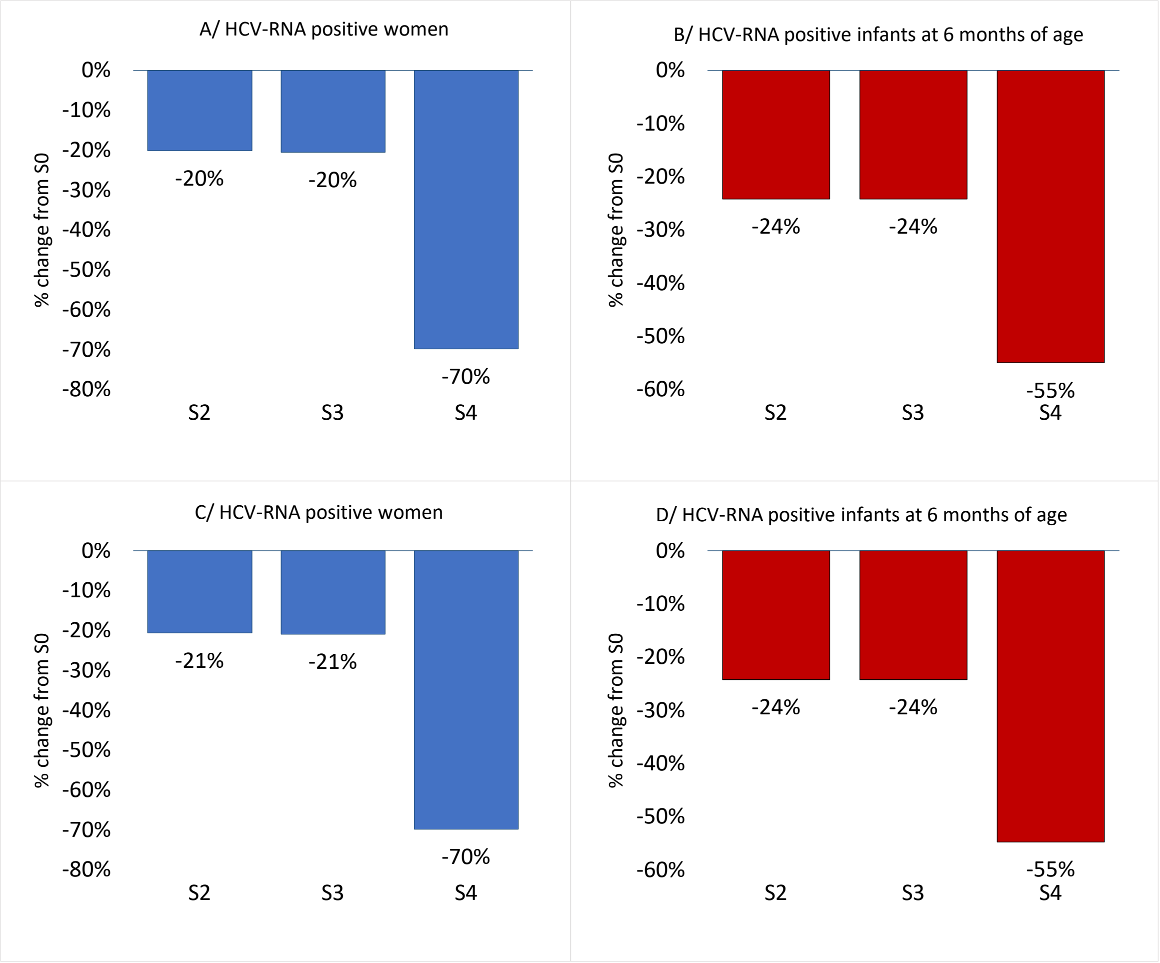

Fig. S12. Sensitivity analysis varying the HCV prevalence among women without HCV risk factors in Ukraine at 0.4% (A, B) and 1.6% (C, D) for the strategies S2 to S4 (S1 has no impact on both outcomes) compared to SOC strategy (S0).

Relative change in proportions of HCV-RNA positive women at the end of pregnancy and in HCV-RNA positive infants at 6 months of age. S0, targeted risk-based screening, no treatment during pregnancy; S2, WHO risk-based screening, targeted DAA during pregnancy; S3, Universal screening, targeted DAA during pregnancy; S4, Universal screening and DAA during pregnancy.

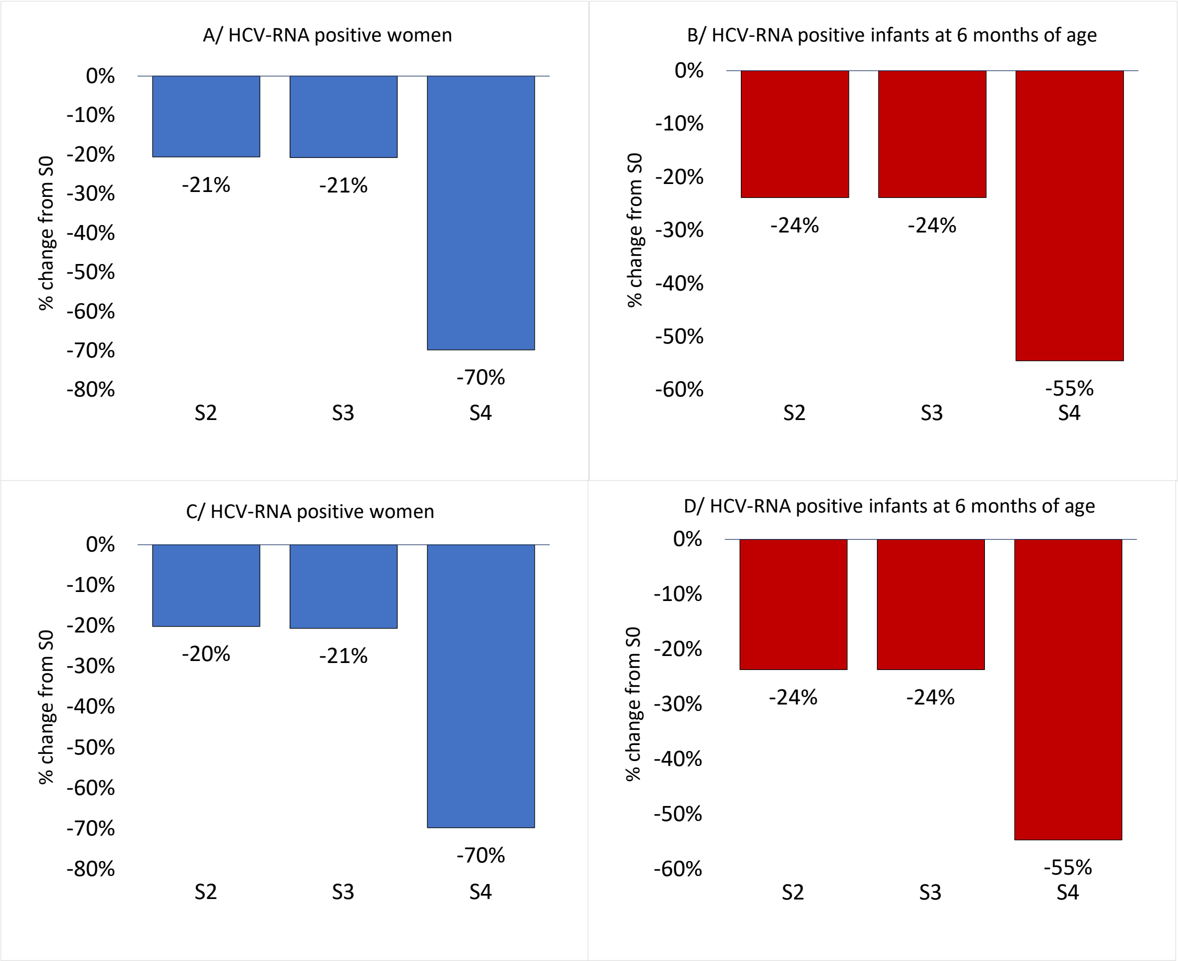

Table S1: Populations with a high HCV prevalence or who have a history of HCV risk exposure/behaviour (24)

Persons who have received medical or dental interventions in health-care settings where infection control practices are substandard
Persons who have received blood transfusions prior to the time when serological testing of blood donors for HCV was initiated or in countries where serological testing of blood donations for HCV is not routinely performed
People who inject drugs (PWID)
Persons who have had tattoos, body piercing or scarification procedures done where infection control practices are substandard
Children born to mothers infected with HCV
Persons with HIV infection
Persons who use/have used intranasal drugs
Prisoners and previously incarcerated persons

Table S2. Model parameter values for both countries (complete version of table 2)

	Egypt	Ukraine	Sources
Number of pregnant women in one year	3,000,000	570,000	(1,4,5,20)
HIV prevalence, % (range)	0.015 (0.014-0.016)	1.0 (0.8-1.2)	(7,8)
Viral suppression among HIV infection (viral load <1000 copies/mL), % (range)	24 (22-26)	86 (68-88)	(7–10)
Presence of at least one HCV risk factor among HIV-negative, % (range)	89 (87-91)	79 (71-87)	(12,25)
Prevalence of HCV-RNA in the presence of at least one HCV infection risk factor, % (range)			
HIV-positive	1.4 (1.3-1.5)	27 (25-29)	(12,14,15)
HIV-negative	1.4 (1.3-1.5)	2.7 (1.6-3.8)	(12,15)
Prevalence of HCV-RNA in the absence of any HCV risk factor, % (range)	0.8 (0.7-0.9)	1 (0.4-1.6)	(12,15)
Prevalence of high HCV viral load (≥ 6log/mL) among HCV-RNA positive, % (range)			

HIV-positive	44 (30-59)		44 (30-59)	(26)
HIV-negative	28 (19-37)		28 (19-37)	
% of women attending ANC at				(2,6)
3 months	77.9		85.3	
5 months	89.7		97.1	
7 months	92.5		99.0	
8 months	93.4		99.3	
9 months	100		100	
C-section, %	44		12	(16,17)
Background maternal mortality per 100,000 live births	37		19	(1)
Fetal loss	HCV-negative	HCV-RNA positive		(3,5)
Miscarriage (1-6 months)	19%	22%	19%	
Stillbirths (>6 months)	4%	11%	1%	

% of women who give birth at		Unsuppressed HIV	Suppressed HIV/HIV-negative	(18,19)
6 months	0·4%	2·4%	1·1%	
7 months	1·2%	7·3%	3·2%	
8 months	4·4%	26·6%	11·9%	
9 months	100%	100%	100%	

Table S3. Model parameter values, range for the univariate sensitivity analysis and distributions used in the probabilistic sensitivity analysis

Parameter	Base case value	Range	Distribution	Distribution parameters	References/Details
Egypt					
HIV prevalence	0.015%	0.014%-0.016%	Beta	α=600; β=4000465	(7,27) Prevalence and its range were calculated from number of women aged 15 and over living with HIV and its interval (5000 [4600-5400]) and general population size in the same age interval (N=33,668,018)
Proportion of HIV-positive women with viral suppression	24%	22%-26%	Beta	α=75; β=239	(7,10) Proportions calculated by multiplying ARV coverage for

					PMTCT (27% [24-29%]) by the 90% of HIV suppressed
Proportion of pregnant women having at least one HCV risk factor	89%	87%-91%	Beta	α=1110; β=137	(12) Base case value obtained from El-Kamary et al and range calculated from a normal distribution assumption.
Prevalence of HCV RNA among population having at least one HCV infection risk factor	1.4%	1.3%-1.5%	Beta	α=98; β=6943	(13) Base case value obtained from Dugan et al. and range calculated by varying +/- 10% the base case value
Prevalence of HCV RNA among population without HCV infection risk factor	0.8%	0.7%-0.9%	Beta	α=99; β=12299	(13) Base case value obtained from Dugan et al and range calculated by varying +/- 10% the base case value
Prevalence of high HCV viral load (≥ 6log/mL) among HCV-RNA positive					(26) Base case value calculated from individual data from Mariné-Barjoan

					et al study cohort and range calculated from a normal distribution assumption
HIV-positive	44%	30%-59%	Beta	α=19; β=24	
HIV-negative	28%	19%-37%	Beta	α=25; β=65	
Proportion of c-section	44%	43%-45%			(16) Base case value obtained from Mostafa et al and range calculated from a normal distribution assumption.
Uptake of HCV screening in S0 when presence of HCV risk factors, and planned c-section	60%	54%-66%	Uniform	54%-66%	Base case value obtained from expert's opinion; range obtained varying +/-10% base case value

Uptake of HCV screening in S0 when presence of HCV risk factors, and no c-section	30%	27%-33%	Uniform	27%-33%	Base case value obtained from expert's opinion; range obtained varying +/-10% base case value
Uptake of HCV screening in S0 in absence of HCV risk factors, and planned c-section	30%	27%-33%	Uniform	27%-33%	Base case value obtained from expert's opinion; range obtained varying +/-10% base case value
Uptake of HCV screening in S0 in absence of HCV risk factors, and no c-section	10%	9%-11%	Uniform	9%-11%	Base case value obtained from expert's opinion; range obtained varying +/-10% base case value
Treatment uptake	78%	70%-86%	Uniform	70%-86%	(25) Base case value obtained from acceptability survey; range obtained varying +/-10% base case value
Proportion of rapid viral response	95%	92%-98%	-	-	(28,29)

Impact of the treatment initiation on VT reduction					
Treatment initiated at least 4 weeks before delivery	80%	72%-80%	Uniform	72%-80%	Assumptions
Treatment initiated less than 4 weeks before delivery	5%	0%-5%	Uniform	0%-5%	
Ukraine					
HIV prevalence	1.0%	0.8%-1.2%	Beta	α=95; β=9419	(8) Prevalence and its ranges were obtained among women aged 15 to 49
Proportion of HIV-positive women with viral suppression	86%	68%-88%	Beta	α=95; β=9419	(8,10) Proportions calculated by multiplying ARV coverage for PMTCT (95 [75-98]) by the 90% of HIV suppressed

Proportion of pregnant women having at least one HCV risk factor	79%	70%-88%	Beta	α=66; β=17	(25) Base case value obtained from acceptability survey; range obtained varying +/- base case value
Prevalence of HCV RNA among HIV-positive women	27%	25%-29%	Beta	α=553; β=1495	(14) Base case value obtained from 33% of pregnant women having HCV ab+ (677 among the population of 2070) that we multiplied by 82% of viraemic and range calculated from a normal distribution assumption.
Prevalence of HCV RNA in the presence of at least one HCV infection risk factor among HIV-negative women	2.7%	1.6%-3.8%	Beta	α=24; β=874	(15) Base case value obtained from 3.6% HCV ab+ in the general population of which 74% are assumed viraemic and range

					calculated from a normal distribution assumption.
Prevalence of HCV RNA in the absence of at least one HCV infection risk factor	1.0%	0.4%-1.6%	Beta	α=11; β=1173	(15) Base case value obtained from 1.3% HCV ab+ in the blood donor population of which 74% are assumed viraemic and range calculated from a normal distribution assumption.
Prevalence of high HCV viral load (≥ 6log/mL) among HCV-RNA positive					(26) Base case value calculated from individual data from the Mariné-Barjoan et al study cohort and range calculated from a normal distribution assumption.
HIV-positive	44%	30%-59%	Beta	α=19; β=24	
HIV-negative	28%	19%-37%	Beta	α=25; β=65	

Proportion of c-section	12%	11%-13%	-	-	(17) Base case value obtained from UNICEF; range obtained varying +/-10% base case value
Uptake of HCV screening in S0 when HIV-positive	87%	78%-96%	Uniform	78%-96%	(25) Base case value obtained from acceptability survey; range obtained varying +/-10% base case value
Uptake of HCV screening in S0 in absence of HCV risk factors	79%	71%-87%	Uniform	71%-87%	(25) Base case value obtained from acceptability survey; range obtained varying +/-10% base case value
Uptake of HCV screening in S0 when presence of HCV risk factors, HIV-negative	75%	68%-83%	Uniform	68%-83%	(25) Base case value obtained from acceptability survey; range obtained varying +/-10% base case value

Treatment uptake	78%	70%-86%	Uniform	70%-86%	(25) Base case value obtained from acceptability survey; range obtained varying +/-10% base case value
Proportion of rapid viral response	95%	92%-98%	-	-	(28,29)
Impact of the treatment initiation on VT reduction					Assumptions
Treatment initiated at least 4 weeks before delivery	80%	72%-80%	Uniform	72%-80%	
Treatment initiated less than 4 weeks before delivery	5%	0%-5%	Uniform	0%-5%	

Supplementary references

1. WHO. Trends in maternal mortality. Geneva: 2019. https://apps.who.int/iris/bitstream/handle/10665/327596/WHO-RHR-19.23-eng.pdf?sequence=13&isAllowed=y (accessed June 07, 2022).

2. Ministry of Health and Population, The DHS program. Egypt Demographic Health Survey 2014. Rockville: 2015. https://dhsprogram.com/pubs/pdf/fr302/fr302.pdf (accessed June 07, 2022).

3. Rezk M, Omar Z. Deleterious impact of maternal hepatitis-C viral infection on maternal and fetal outcome: a 5-year prospective study. Arch Gynecol Obstet. 2017 Dec;296(6):1097–102.

4. Smits J, Monden C. Twinning across the Developing World. Newell ML, editor. PLoS ONE. 2011 Sep 28;6(9):e25239.

5. Stover J, Winfrey W. The effects of family planning and other factors on fertility, abortion, miscarriage, and stillbirths in the Spectrum model. BMC Public Health. 2017 Nov;17(S4):775.

6. Ministry of Health, The DHS program. Calverton: 2008. Ukraine Demographic and Health Survey 2007. https://dhsprogram.com/pubs/pdf/FR210/FR210.pdf (accessed June 07, 2022).

7. UNAIDS. Women aged 15 to 49 HIV prevalence rate, Egypt. 2020. https://www.unaids.org/fr/regionscountries/countries/egypt (accessed June 07, 2022).

8. UNAIDS. Women aged 15 to 49 HIV prevalence rate, Ukraine. 2020. https://www.unaids.org/fr/regionscountries/countries/ukraine (accessed June 07, 2022).

9. Yotebieng M, Mpody C, Ravelomanana NL, Tabala M, Malongo F, Kawende B, et al. HIV viral suppression among pregnant and breastfeeding women in routine care in the Kinshasa province: a baseline evaluation of participants in CQI-PMTCT study. J Int AIDS Soc. 2019 Sep;22(9).

10. Kintu K, Malaba TR, Nakibuka J, Papamichael C, Colbers A, Byrne K, et al. Dolutegravir versus efavirenz in women starting HIV therapy in late pregnancy (DolPHIN-2): an open-label, randomised controlled trial. Lancet HIV. 2020 May;7(5):e332–9.

11. Snijdewind IJM, Smit C, Schutten M, Nellen FJB, Kroon FP, Reiss P, et al. Low mother-to-child-transmission rate of Hepatitis C virus in cART treated HIV-1 infected mothers. J Clin Virol. 2015 Jul;68:11–5.

12. El-Kamary SS, Hashem M, Saleh DA, Ehab M, Sharaf SA, El-Mougy F, et al. Reliability of risk-based screening for hepatitis C virus infection among pregnant women in Egypt. J Infect. 2015 May;70(5):512–9.

13. Dugan E, Blach S, Biondi M, Cai Z, DePaola M, Estes C, et al. Global prevalence of hepatitis C virus in women of childbearing age in 2019: a modelling study. Lancet Gastroenterol Hepatol. 2021 Mar;6(3):169–84.

14. Bailey H, Nizova N, Martsynovska V, Volokha A, Malyuta R, Cortina-Borja M, et al. HCV co-infection and markers of liver injury and fibrosis among HIV-positive childbearing women in Ukraine: results from a cohort study. BMC Infect Dis. 2016 Dec 12;16(1):755.

15. Hope VD, Eramova I, Capurro D, Donoghoe MC. Prevalence and estimation of hepatitis B and C infections in the WHO European Region: a review of data focusing on the countries outside the European Union and the European Free Trade Association. Epidemiol Infect. 2014 Feb;142(2):270–86.

16. Mostafa A, Ebeid FSE, Khaled B, Ahmed RHM, El-Sayed MH. Micro-elimination of hepatitis C through testing of Egyptian pregnant women presenting at delivery: implications for screening policies. Trop Med Int Health. 2020;25(7):850–60.

17. Unicef. Key demographic indicators, Ukraine. https://data.unicef.org/country/ukr/ (accessed June 07, 2022).

18. Statista. Distribution des femmes ayant accouché en France en 2016, selon l'âge gestationnel à la naissance du bébé. https://fr.statista.com/statistiques/800059/grossesse-femmes-age-gestationnel-accouchement-france/.

19. Albert AYK, Elwood C, Wagner EC, Pakzad Z, Chaworth-Musters T, Berg K, et al. Investigation of factors associated with spontaneous preterm birth in pregnant women living with HIV: AIDS. 2020 Apr;34(5):719–27.

20. WHO World Health Organization. Maternal mortality in 2000-2017 in Ukraine. [Cited 05 October 2021] Available from: https://www.who.int/gho/maternal_health/countries/ukr.pdf?ua=1.

21. Ades AE, Gordon F, Scott K, Collins IJ, Thorne C, Pembrey L, et al. Overall vertical transmission of HCV, transmission net of clearance, and timing of transmission. Clin Infect Dis. (Online ahead of print);

22. Ades AE, Gordon F, Scott K, Collins IJ, Thorne C, Pembrey L, et al. Spontaneous clearance of vertically acquired hepatitis c infection: implications for testing and treatment. Clin Infect Dis. (Online ahead of print);

23. Briggs AH, Weinstein MC, Fenwick EAL, Karnon J, Sculpher MJ, Paltiel AD. Model Parameter Estimation and Uncertainty Analysis: A Report of the ISPOR-SMDM Modeling Good Research Practices Task Force Working Group–6. Med Decis Making. 2012 Sep;32(5):722–32.

24. WHO. Guidelines for the screening, care and treatment of persons with chronic hepatitis C infection, Updated version April 2016. Geneva: World Health Organization, 2016. https://apps.who.int/iris/handle/10665/205035 (accessed June 01, 2022).

25. Scott K, Chappell E, Mostafa A, Volokha A, Najmi N, Ebeid F, et al. [Preprint] Acceptability of Hepatitis C screening and treatment during pregnancy in pregnant women in Egypt, Pakistan and Ukraine [Internet]. 2021 Sep. Available from: http://medrxiv.org/lookup/doi/10.1101/2021.09.29.21264171 (accessed June 07, 2022)

26. Mariné-Barjoan E, Berrébi A, Giordanengo V, Favre SF, Haas H, Moreigne M, et al. HCV/HIV co-infection, HCV viral load and mode of delivery: risk factors for mother-to-child transmission of hepatitis C virus? AIDS. 2007 Aug 20;21(13):1811–5.

27. Population Pyramid. Population pyramid of Egypt 2020. [cited 17 March 2022]; Available from: https://www.populationpyramid.net/egypt/2020/.

28. Feld JJ, Jacobson IM, Hézode C, Asselah T, Ruane PJ, Gruener N, et al. Sofosbuvir and Velpatasvir for HCV Genotype 1, 2, 4, 5, and 6 Infection. N Engl J Med. 2015 Dec 31;373(27):2599–607.

29. Foster GR, Afdhal N, Roberts SK, Bräu N, Gane EJ, Pianko S, et al. Sofosbuvir and Velpatasvir for HCV Genotype 2 and 3 Infection. N Engl J Med. 2015 Dec 31;373(27):2608–17.
